# A Biodegradable Radical Polymer Enables High‐Performance, Physically Transient Organic Memory

**DOI:** 10.1002/anie.202422826

**Published:** 2025-05-12

**Authors:** Jaehyoung Ko, Soeun Kim, Daeun Kim, Taeho Lim, Soyeong Jin, Youngdo Jeong, Yongho Joo, Sangho Cho

**Affiliations:** ^1^ Functional Composite Materials Research Center Korea Institute of Science and Technology Jeonbuk 55324 Republic of Korea; ^2^ Extreme Materials Research Center Korea Institute of Science and Technology Seoul 02792 Republic of Korea; ^3^ School of Materials Science and Engineering Gwangju Institute of Science and Technology Gwangju 61005 Republic of Korea; ^4^ Center for Advanced Biomolecular Recognition Biomedical Research Division Korea Institute of Science and Technology Seoul 02792 Republic of Korea; ^5^ Department of Chemistry Hanyang University Seoul 04763 Republic of Korea; ^6^ Department of HY‐KIST Bio‐convergence Hanyang University Seoul 04763 Republic of Korea; ^7^ Department of Nanoscience and Technology, KIST School Korea University of Science and Technology Seoul 02792 Republic of Korea

**Keywords:** Biodegradable polymers, Organic memristors, Physically transient electronics, Radical polymers, Soft bioelectronics

## Abstract

Electronic devices often demand high reliability and longevity, but they also contribute significantly to electronic waste. Physically transient electronics have thus emerged as a promising alternative in future electronics, particularly in wearable and implantable bioelectronics. In these applications, memristive materials have gained significant attention for their potential to realize neuromorphic systems that offer energy‐efficient, hardware‐based parallel processing. By integrating memristive capabilities with transient behavior, this study bridges these two cutting‐edge fields, creating materials that not only enable advanced computing but also dissociate sustainably. Additionally, we leverage the unique features of soft materials for their tunability, biocompatibility, and cost‐effectiveness, which collectively enhance this integration. In this work, we first illustrate molecular engineering strategy on a radical polymer. We then proceed to two‐terminal devices therefrom, which exhibit exceptional memory performance of >10^6^ on/off ratio, >10^4^ s state retention, and stability over 250 DC sweep cycles. A flexible, optically transparent, and physically transient crossbar arrays are also developed, which maintain the performance through >3,000 bending cycles and fully dissociate in water at room temperature. This work represents an advancement toward a biorealistic platform with substantial multifunctionality, making it readily translatable to future wearable and implantable neuromorphic devices.

## Graphical abstract

A stable radical polymer with intrinsic memristivity and on‐demand physical transience is presented. We leverage the nonconjugated nature of the radical polymer for a systematic molecular engineering. The polymer enabled a high‐performance organic memristive device comparable to inorganic transient memory devices, now equipped with additional functionalities including optical transparency, flexibility, biocompatibility, and the transience.

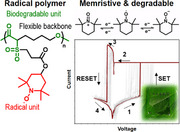

## Introduction

Electronic devices provide substantial utility and convenience in modern society. In particular, the demand for reliable and long‐lasting devices has deeply motivated both industry and academia in the pursuit of next‐generation electronics.^[^ ^1^, ^2^, ^3^ ^]^ Yet, device longevity is not always the best solution;^[^ ^4^, ^5^, ^6^ ^]^ For example, persistent electronic wastes (e‐wastes) and plastics are causing serious environmental problems, posing global concerns for wildlife and public health.^[^ ^7^, ^8^ ^]^ Physically transient electronics, where all or part of the device can dissociate or degrade on demand, therefore present significant future opportunities as per their green chemistry, cost‐effectiveness, and target‐specific transience.^[^ ^4^, ^9^, ^10^ ^]^ Importantly, they align well with the emerging flexible and wearable electronics^[^ ^11^, ^12^, ^13^, ^14^ ^]^ and are expected to be compatible with future bioelectronics in general.

Memristive materials have emerged as a promising candidate for realizing hardware‐based neuromorphic computing systems, offering minimal energy consumption through massive parallelism in data processing.^[^ ^15^, ^16^ ^]^ Among these, organic memristive materials provide additional advantages, including molecular tunability, material compliance, lightweight properties, and cost‐effectiveness.^[^ ^17^, ^18^ ^]^ Moreover, their inherent biocompatibility facilitates seamless integration into biological systems with minimal personal or electrical disturbance.^[^ ^6^, ^19^ ^]^ These characteristics collectively enable the development of “soft” biorealistic architectures, such as human body‐computer integration or implantable neuromorphic devices.

While the aforementioned features of an advanced form of biorealistic systems present substantial technological and societal impacts, their practical realization has rarely been achieved or successfully reproduced to date.^[^ ^4^, ^10^, ^20^ ^]^ These systems, in particular, hold significant promise in areas such as (1) patient‐friendly medical diagnostics and personalized therapy, (2) environmental sustainability and e‐waste reduction, and (3) next‐generation human‐machine interfaces.^[^ ^4^ ^]^ A key challenge lies in developing a versatile material platform capable of integrating these multifunctionalities into one; Such material platform should ideally possess: (i) high electrical performance and tunability (or memristivity), (ii) controlled (bio‐) degradability or target specific (physical) transience, (iii) mechanical compliance or processability with minimal operational disturbance, (iv) and cost‐effectiveness in terms of both the data processing and the device fabrication, preferably all at the same time.

Among these challenges, achieving both high device performance and functionality has been particularly difficult due to the general trade‐off between the two.^[^ ^10^ ^]^ This has particularly been the case for memory architectures equipped with the physical transience. For example, they have often adopted biomaterials as active layers that are neither intrinsically memristive nor electrically active.^[^ ^21^, ^22^ ^]^ In such cases, memristivity was imparted from other device components, such as electrochemical metallization of electrodes. Alternatively, some incorporated metallic nanoparticles as additives to enable the memory function.^[^ ^23^ ^]^ In contrast, the devices based on inorganic memristors have typically exhibited limited physical transience and biocompatibility.^[^ ^4^ ^]^ Overall, the current state of soft bioelectronics importantly calls for a novel material platform that effectively break through the aforementioned limitations.

In our previous work, we demonstrated that a nonconjugated open‐shell polymer (i.e., radical polymer) is intrinsically memristive and capable of replicating synaptic cooperativity observed in biological systems.^[^ ^24^ ^]^ Notably, the nonconjugated nature of the radical polymer allows for additional degree of freedom to the molecular engineering, potentially providing researchers with a vast library of tunable functionalities that are not easily achievable in conjugated organics or inorganic memristive materials.^[^ ^25^ ^]^ As a logical next step toward molecular engineering that bridges the two intimate fields of research, we demonstrate that equipping this system with on‐demand physical transience enables the development of an advanced computing platform with target‐specific transience and environmental sustainability.

In this work, we present a nonconjugated radical polymer capable of memristive function and controlled dissociation as a single‐component material for the realization of the soft biorealistic systems. Specifically, we first outline the synthetic strategies employed to enable systematic molecular engineering of a prototype radical polymer. We then conduct a detailed investigation on the physical and electrical properties of the memristive device therefrom. Notably, we report exceptional memory performance including an on/off ratio of >10^6^, state retention exceeding 10^4^ seconds, and device stability after more than 250 DC sweep cycles. The crossbar array displayed high optical transparency, flexibility, and the physical transience at device performance well exceeding other transient memristors reported so far. Furthermore, the array demonstrated stable device operation through over 3000 bending cycles and displayed a full dissociation under mild conditions in pure water at room temperature. We believe our work demonstrates an advanced soft biorealistic platform, which can readily be adapted into wearable and implantable neuromorphic devices in various form factors.

## Results and Discussion

**Figure 1:**
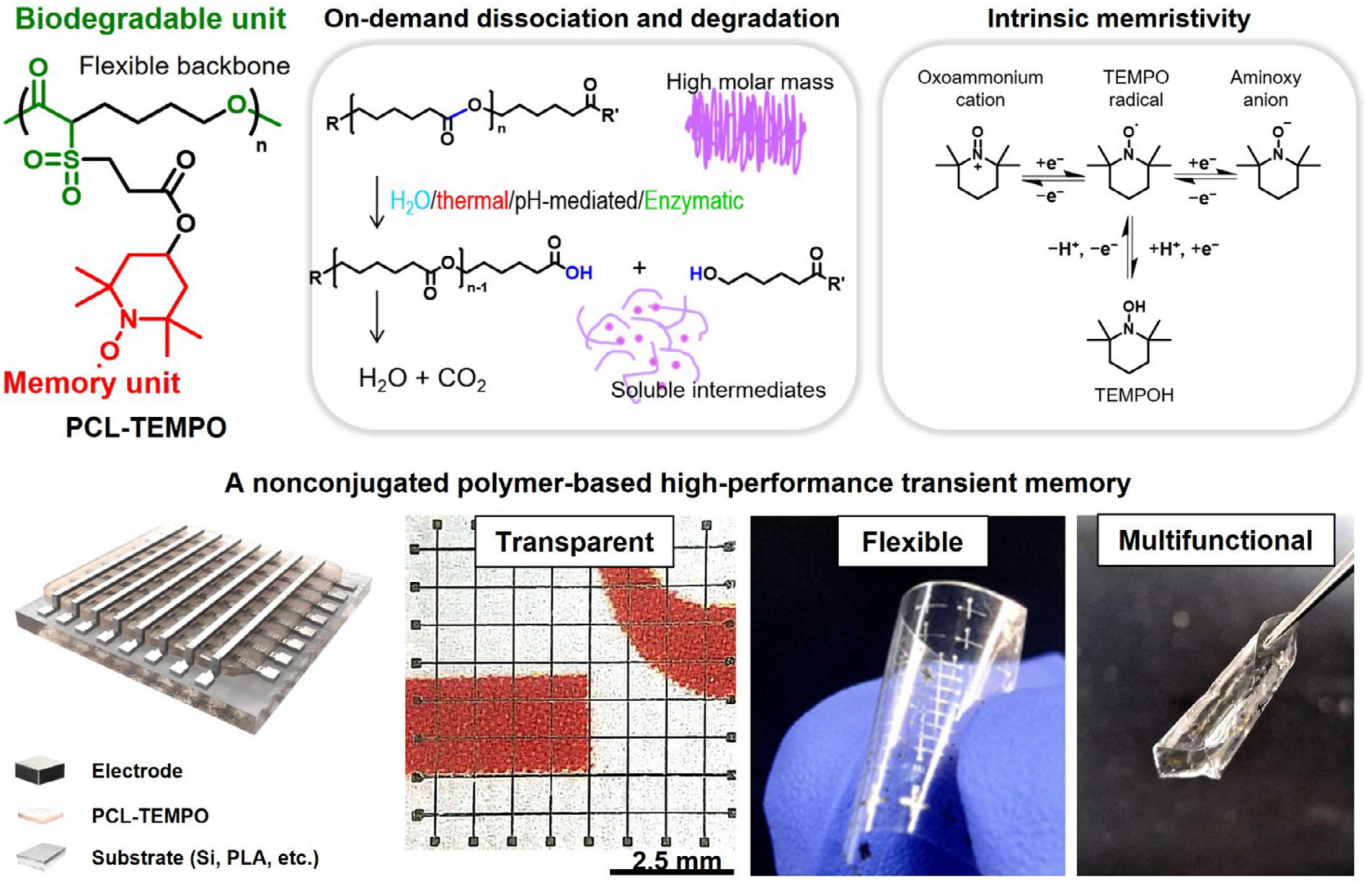
Schematic illustration of a nonconjugated polymer‐based high‐performance transient memory.

The radical polymer used in this study, designed to be both intrinsically memristive and physically transient, is a nitroxide‐based radical polymer derivative, poly(ε‐caprolactone 2,2,6,6‐tetramethylpiperidin‐1‐yl) (**PCL‐TEMPO**) (Figure 1). The polymer is conductive due to the carrier hopping between active radical sites.^[^ ^25^, ^26^ ^]^ The pendant radical exhibits intrinsic memristivity from its variable redox states (and thus the conductance states).^[^ ^24^ ^]^ The caprolactone linkage in the main chain enables on‐demand physical transience of the polymer. Additionally, the nonconjugated hydrocarbon backbone imparts overall flexibility and optical transparency to the polymer (Figure 1, top panel).^[^ ^27^, ^28^ ^]^ The polymer can serve as an active layer in a two‐terminal device (i.e., resistor), where distinct conductance states are achieved in a nonvolatile manner to store information bits. Notably, a crossbar‐type array of such devices can naturally enable a neuromorphic computing architecture, allowing for the realization of a hardware‐based biorealistic system.^[^ ^15^ ^]^ Such soft neuromorphic system has immediate applications in implantable neuromorphic devices, where memory performance, optical transparency, flexibility, and the physical transience are required at the same time (Figure 1, bottom panel).

### Synthesis of PCL‐TEMPO

The nonconjugated nature of a radical polymer allows for facile molecular engineering to achieve specific end‐use properties. For example, in our previous work, we incorporated ethylene oxide into the backbone of a radical polymer, significantly enhancing its ionic conductivity and, consequently, its mixed conductivity by up to three orders of magnitude.^[^ ^24^ ^]^ Our focus here centers on the synthetic tunability, flexibility, biocompatibility, and potential physical transience of polycaprolactones (PCLs), which, while functioning orthogonally, synergistically enable a transient memristor when combined with the intrinsic memristivity of a radical polymer.

**Figure 2:**
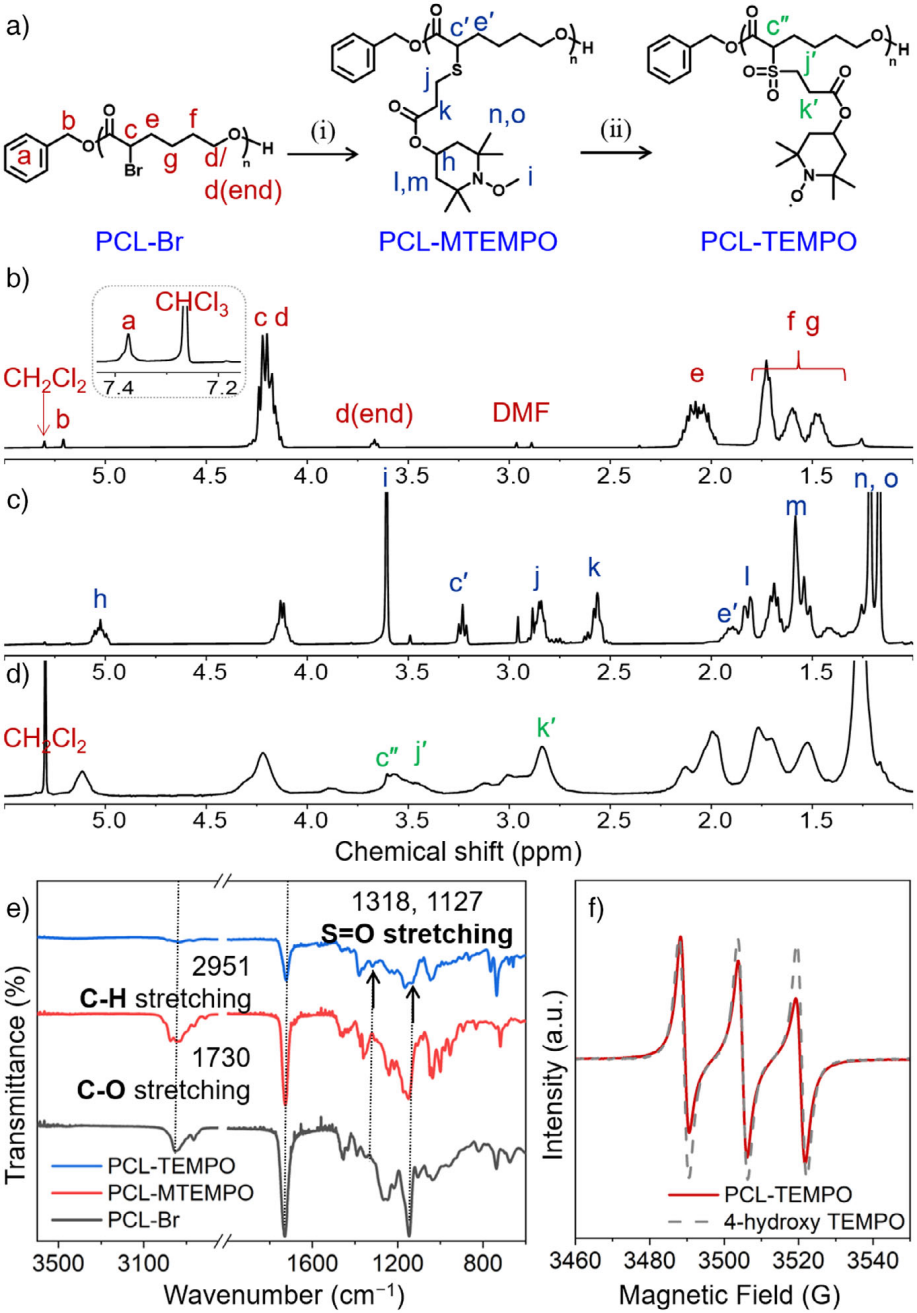
Synthesis of PCL‐TEMPO. a) Scheme for the synthesis of PCL‐TEMPO: (i) thio‐MTEMPO, Et_3_N, DMF, RT, and 4 h, and (ii) *m*CPBA, DCM, 0 °C to RT, and 4 h. b)–d) ^1^H NMR spectra (CDCl_3_, 400 MHz) of (b) PCL‐Br, (c) PCL‐MTEMPO, and (d) PCL‐TEMPO. e) FTIR spectra of the reaction products, and f) ESR spectra of PCL‐TEMPO (solid line) and 4‐hydroxy TEMPO (dashed line) in solution.

The synthesis of **PCL‐TEMPO** proceeds through the ring‐opening of an ε‐caprolactone derivative, followed by the click attachment of a radical moiety (TEMPO, 2,2,6,6‐tetramethylpiperidin‐1‐yl) as a pendant unit (Figure 2a). The monomer, 2‐bromo‐caprolactone (hereafter referred to as CL‐Br) was used to incorporate the caprolactone linkage throughout the main chain and equip the resulting polymer with a redox‐active unit. Thio‐MTEMPO (1‐methoxy‐2,2,6,6‐tetramethylpiperidin‐4‐yl 3‐mercaptopropanoate) and CL‐Br were prepared according to literature procedures, as described in Supporting Information (Scheme S1) with ^1^H and ^13^C NMR analysis (Figures S1 to S9).^[^ ^29^, ^30^ ^]^ The polymeric intermediate (hereafter referred to as **PCL‐Br**) was obtained as a linear homopolymer with a low dispersity (*Ð*) value of 1.12 and a number‐averaged molecular weight (*M*_n_) of 8.8 kDa. Specifically, the reaction occurred in the presence of benzyl alcohol (BnOH) as an initiator, and stannous octoate (Sn(Oct)_2_) as a catalyst (feed ratio, [CL‐Br]:[BnOH]:[Sn(Oct)_2_] = 55:1:0.17, 130 °C, 3 h). The degree of polymerization (*DP*) was determined by ^1^H NMR, yielding an integral ratio of 43 (Figure 2b, between the methylene protons at 2.07 (**e**) and 3.66 (**d** end) ppm).

To introduce a stable radical moiety in the biodegradable polymer backbone, **PCL‐Br** was functionalized with thio‐MTEMPO in DMF via thio‐bromo click reaction at room temperature using triethylamine (Et_3_N) as a catalyst.^[^ ^31^ ^]^ Thio‐MTEMPO with a protected radical group was prepared to prevent undesired interaction between the nitroxide and thiol groups as the click reaction is typically radical‐mediated (Figure S1). ^1^H NMR spectrum of **PCL‐Br** (Figure 2b) showed that the peak at 4.19 ppm (**c** and **d**), which was assigned to the methine proton adjacent to the Br and methylene protons adjacent to the O atom, respectively. After the protected radical moieties were attached to **PCL‐Br** (hereafter referred to as **PCL‐MTEMPO**), the methine proton adjacent to Br group showed an NMR shift to 3.23 ppm (**c′**) via formation of thioether bond. The newly appeared peaks at 5.17, 3.60, 2.85, 2.56,1.81, 1.58, 1.21, and 1.17 ppm were attributed to the protons of thio‐MTEMPO (Figure 2c). In ^13^C NMR spectra (Figure S9), the peak at 45.73 ppm of the carbon adjacent to the Br group in **PCL‐Br** was shifted to 46.67 ppm after the converting to thioether bond in **PCL‐MTEMPO**. In the ^1^H NMR spectrum of **PCL‐MTEMPO**, the integral ratio of peaks **c′** and **j** was 1:2, confirming that thio‐MTEMPO was completely introduced to the **PCL‐Br** as a pendant group. The profiles of size exclusion chromatography (SEC) also demonstrated the successful functionalization, showing an *M*_n_ of 14.5 kDa, with the *Ð* of 1.24 (Figure S10). **PCL‐MTEMPO** was then oxidized with *meta*‐chloroperoxybenzoic acid (*m*CPBA) in anhydrous methylene chloride to generate **PCL‐TEMPO**. ^1^H NMR spectrum (Figure 2d) confirmed that the methyl group on the nitroxide in **PCL‐MTEMPO** was removed by observing disappearance of a sharp peak (**i**) at 3.6 ppm, representing complete conversion to the active radical moiety. Line broadening indicates the presence of a stable nitroxide radical, which interacts with nearby nuclear spins and accelerates the relaxation.^[^ ^32^ ^]^ The nitroxide group in **PCL‐TEMPO** can assume variable redox states, with the representatives being a neutral nitroxide (NO^•^) and its oxidized form (oxoammonium, NO^+^).^[^ ^29^, ^31^ ^]^ With Fourier transform infrared (FTIR) spectroscopy, we investigated on the redox states, based on the distinct absorption of the oxoammonium species at 1540 cm^−1^.^[^ ^25^, ^33^ ^]^ In our case, no peak was detected from 1500 to 1600 cm^−1^ as shown in Figure 2e, confirming that **PCL‐TEMPO** contains negligible amounts of cation species and remains as the neutral form. Another issues associated with the synthesis is the conversion of the sulfide group into sulfone or sulfoxide, at oxidative reaction conditions.^[^ ^31^, ^34^ ^]^ The ^1^H NMR spectrum (Figure 2d) showed that the methine proton (**c″**) adjacent to the sulfone and the α‐position was shifted from 3.23 to 3.61 ppm, and the methylene proton (**j′**) adjacent to other side of the sulfone was shifted from 2.85 to 3.61 ppm. The FTIR spectrum of **PCL‐TEMPO** confirmed the presence of characteristic sulfone peaks at 1318 and 1127 cm^−1^, but no sulfoxide peak at 1018 cm^−1^ as shown in Figure 2e. As a final product, **PCL‐TEMPO** displayed an increased *M*_n_ of 12.4 kDa, and a *Ð* of 1.12 (Table S1). The presence of active radicals was confirmed through electron spin resonance (ESR) analysis, which indicated redox pairs that provide the system with distinct conductance states (Figure 2f). Overall, **PCL‐TEMPO** displayed good solubility in organic solvents including DMF, THF, DCM, and acetone, which is advantageous in the device fabrication that ensues.

### Thermal Properties of PCL‐TEMPO

Building on the successful synthesis of **PCL‐TEMPO**, we conducted a detailed investigation of its thermal property. This aspect is critical for device fabrication and its performance, essential considerations for practical applications in soft electronics. Specifically, since the physical state of the polymer associated with the glass transition temperature (*T*_g_) can influence the quality of the active layer significantly,^[^ ^35^ ^]^ it is often desirable to consider an additional fine‐tuning of the thermal properties. For example, it can be simply achieved through appropriate molecular engineering strategy of controlling the molecular weight of the polymer.

First, thermal stability of **PCL‐TEMPO** and its precursor polymers were studied by thermogravimetric analysis (TGA, Figure S10). The decomposition temperatures at 5 wt% weight loss of the synthesized **PCL‐Br**, **PCL‐MTEMPO**, and **PCL‐TEMPO** were observed at 238, 216, and 166 °C, respectively. The decomposition temperature (*T*_d_) of **PCL‐MTEMPO** was lower than that of **PCL‐Br** due to the lower bond dissociation energy of C–S (259 kJ mol^−1^) than that of C–Br (276 kJ mol^−1^).^[^ ^36^ ^]^ The *T*_d_ of **PCL‐TEMPO** was lowered than that of **PCL‐MTEMPO** due to the presence of sulfone linkage. The *T*_g_ for **PCL‐Br**, **PCL‐MTEMPO**, and **PCL‐TEMPO** were observed at −37.3, −9.7, and 50.9 °C, respectively. **PCL‐Br** showed higher *T*_g_ value but no melting point (*T*_m_), when compared to conventional (semicrystalline) PCL that typically displays *T*_g_ of −62 °C and the *T*_m_ of 5 –65 °C.^[^ ^37^, ^38^, ^39^ ^]^ This is due to the electron withdrawing nature of the bromine, which causes the dipole interaction that results in stronger intermolecular forces than that of PCL. We observed that the *T*_g_ of **PCL‐MTEMPO** increased compared to that of **PCL‐Br**, which is attributed to the bulkiness of MTEMPO that increases overall molecular weight of the polymer and the free volume.^[^ ^40^, ^41^, ^42^ ^]^ Here, the molecular weight is related to the *T*_g_ by the Fox–Flory relation.^[^ ^43^, ^44^ ^]^ After the oxidation, **PCL‐TEMPO** features nitroxides and sulfone groups with its much higher *T*_g_ value. The nitroxide can act as an acceptor of the hydrogen bond, resulting in hydrogen bonded networks.^[^ ^45^ ^]^ The sulfone groups are more rigid compared to sulfides, also leading to the higher *T*_g_ value. As higher *T*_g_ can improve the uniformity of an organic film, that of **PCL‐TEMPO** is expected to achieve generally lower device‐to‐device variability compared to that of the prototype device (in our previous work). Overall, the unique set of physical properties exhibited by **PCL‐TEMPO** strongly indicates its potential toward biointerfaces and bioelectronics.

### Memory Performance of PCL‐TEMPO

**Figure 3:**
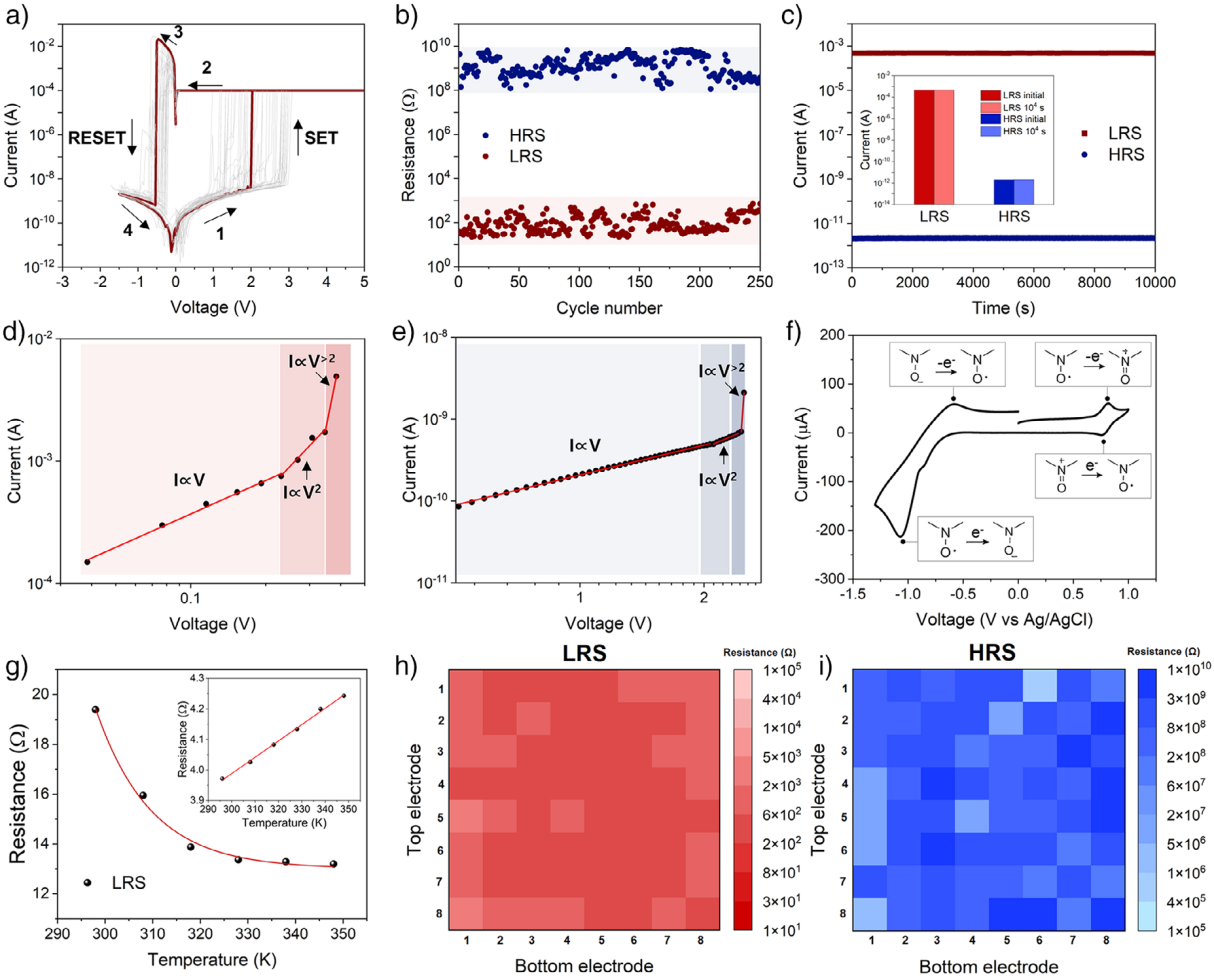
Memory performance of PCL‐TEMPO. a) *I–V* curves of PCL‐TEMPO based MIM device exhibiting reliable switching performance. b) Cycling test displaying on/off ratio >10^6^ over >250 cycles. c) Retention test displaying stable device operation for >10^4^ s. d) and e) Mechanistic analysis on the conduction behaviors of PCL‐TEMPO device at (d) LRS, and (e) HRS. f) Cyclic voltammogram of 0.1 mM PCL‐TEMPO in acetonitrile, with 0.1 M tetrabutylammonium hexafluorophosphate as an electrolyte. g) Temperature dependence of the LRS in a PCL‐TEMPO based device. The inset shows a typical resistance–temperature relationship observed in metals (Au in this case). h) and i) Color map images of resistance distribution from *I–V* curves of 64 memory cells at (h) LRS and (i) HRS.

Based on the successful synthesis and stable thermal properties of **PCL‐TEMPO**, we now explore the intrinsic memory performance of **PCL‐TEMPO** based devices. As mentioned earlier, the successful implementation of **PCL‐TEMPO** in hardware‐based biorealistic systems requires its intrinsic memristivity. To test this possibility, we first constructed metal‐insulator‐metal (MIM) devices using **PCL‐TEMPO** as the active layer, for both dot and crossbar arrays. We utilized gold, copper, and molybdenum as electrodes (30 nm bottom and 50 nm top electrodes), where consistent active layer thickness of 100 nm was applied unless otherwise noted. Regarding electrode selection, we observed similar resistive switching behavior across all electrode combinations, consistent with our previous work.^[^ ^24^ ^]^ However, we recommend using an asymmetric configuration of gold and copper as it provides the most stable resistive switching characteristics presumably due to the favorable energy level alignment (absence of electrochemical metallization is thoroughly investigated below). While the electrode dependence in resistive switching can be a nontrivial issue, a detailed investigation is beyond the scope of this work and is deferred to our ongoing research. For the device area, the dot arrays were tested with areas of 50 × 50, 100 × 100, and 200 × 200 µm^2^, while a fixed area of 50 × 50 µm^2^ was used for the crossbar arrays (further device fabrication details are given in the Experimental Section). Figure 3a summarizes the typical *I–V* sweep of the **PCL‐TEMPO** based MIM device. Measurements were performed in voltage sweeping mode at ambient conditions (i.e., room temperature without applied vacuum). We found that the device exhibited *I–V* curves typical of bipolar resistive switching, with a clear distinction between high resistance states (HRS) and low resistance states (LRS), abrupt transitions between them, and a polarity reversal between the SET and RESET processes. The DC voltage sweep was highly reliable and repeatable, with SET and RESET voltages (*V*_SET_ and *V*_RESET_) occurring at approximately 2.0 V and −0.5 V for both device types. Overall, the device demonstrated an impressive on/off ratio exceeding 10^6^, and it maintained stable performance after more than 250 DC sweep cycles (Figure 3b). In addition, the device exhibited remarkable state retention characteristics that lasts over 10^4^ seconds (Figure 3c).

For comparison, we tested control devices composed of a PCL active layer (i.e., without TEMPO moiety) with varying (i) thickness and (ii) electrode combinations. Specifically, no switching events were observed in devices with either a thinner active layer (50 nm) or an Au (bottom) / Cu (top) electrode configuration even when subjected to an extended applied voltage (Figure S11). Note the operation voltage could be adjusted down to less than 1 V by appropriate device engineering (e.g., tuning of the active layer thickness) (Figure S12). Furthermore, we tested whether the LRS current is externally limited by the measurement setup, where we found no significant change in LRS current across compliance current (CC) values ranging from 10^−3^ to 10^−6^ A per order of magnitude (Figure S13). Last, we present a case study of a 50 nm‐thick **PCL‐TEMPO** device, where the low‐lying LRS current enabled stable resistive switching with no application of CC, without causing permanent damage to the device (Figure S14). Overall, these additional experiments strongly support the molecular origin of the resistive switching in the **PCL‐TEMPO** device system. We also note that the electrical characteristics of **PCL‐TEMPO** is highly competitive to a class of inorganic transient memristors, and greatly surpasses those of organic memristors reported previously (see below).^[^ ^10^ ^]^ As a result, these characteristics highlight the potential of **PCL‐TEMPO** in advanced soft bioelectronics, offering highly stable and reliable performance in terms of both device operation and the material itself.

For further investigation on the origin of the intrinsic memristivity exhibited by **PCL‐TEMPO**, we analyzed the double logarithmic *I–V* curves of a typical switching cycle (Figure 3d,e). Three distinct charge conduction regimes were identified at both HRS and LRS: (i) Ohmic conduction (*I*∝*V*), (ii) square law dependence (*I*∝*V* ^2^), and (iii) regions of steep current increase (*I*∝*V* ^α^ with α > 2). This indicates the space‐charge limited conduction (SCLC) dominates both conductance states, and thus rules out the possibility of resistive switching caused by metallic filament formation that typically shows Ohmic conduction across the voltage sweep.^[^ ^46^, ^47^ ^]^ Additionally, to investigate the origin of the bistability observed in the *I–V* measurements, cyclic voltammetry (CV) was performed. Figure 3f shows a typical voltammogram of 0.1 mM **PCL‐TEMPO** in acetonitrile, in the presence of 0.1 M tetrabutylammonium hexafluorophosphate. The voltammogram closely aligned with those of small‐molecule nitroxides reported in the literature,^[^ ^48^ ^]^ displaying reversible and stable redox kinetics within a given voltage window. Specifically, oxidation peaks were observed at ∼ 0.7 V (vs. Ag/AgCl), and reduction peaks occurred at ∼ −1 V (vs. Ag/AgCl). Further, we examined the temperature dependence of the LRS current as radical polymers typically exhibit a systematic decrease in resistance with increasing temperature.^[^ ^25^, ^26^ ^]^ This behavior contrasts sharply with resistive switching devices based on the electrochemical metallization mechanism, which generally show linear increase in the resistance as temperature increases. Following the LRS resistance test, we observed a systematic decrease in resistance in **PCL‐TEMPO** based devices as temperature increases, further corroborating the molecular origin of our device system (Figure 3g). Although the experimental evidence on the molecular origin of the resistive switching observed in our system is self‐supporting, we refer readers to our previous efforts on prototype radical polymer‐based resistive switching devices.^[^ ^24^ ^]^ Lastly, as device yield and variability remain critical challenges in both inorganic and organic memristive systems, we conducted a statistical analysis of our 8 × 8 crossbar array (Figure 3h,i). Our results revealed a remarkably high device yield but significant device‐to‐device variability in both HRS and LRS currents. We anticipate that future studies and further optimization of the device fabrication process will help minimize this variability, which we aim to address in our ongoing research. Overall, the mechanistic studies, together with electrochemical measurements, confirm that the intrinsic memristivity of **PCL‐TEMPO** that can be utilized in soft biorealistic systems.

### Designer Features of PCL‐TEMPO Toward Soft Bioelectronics

**Figure 4:**
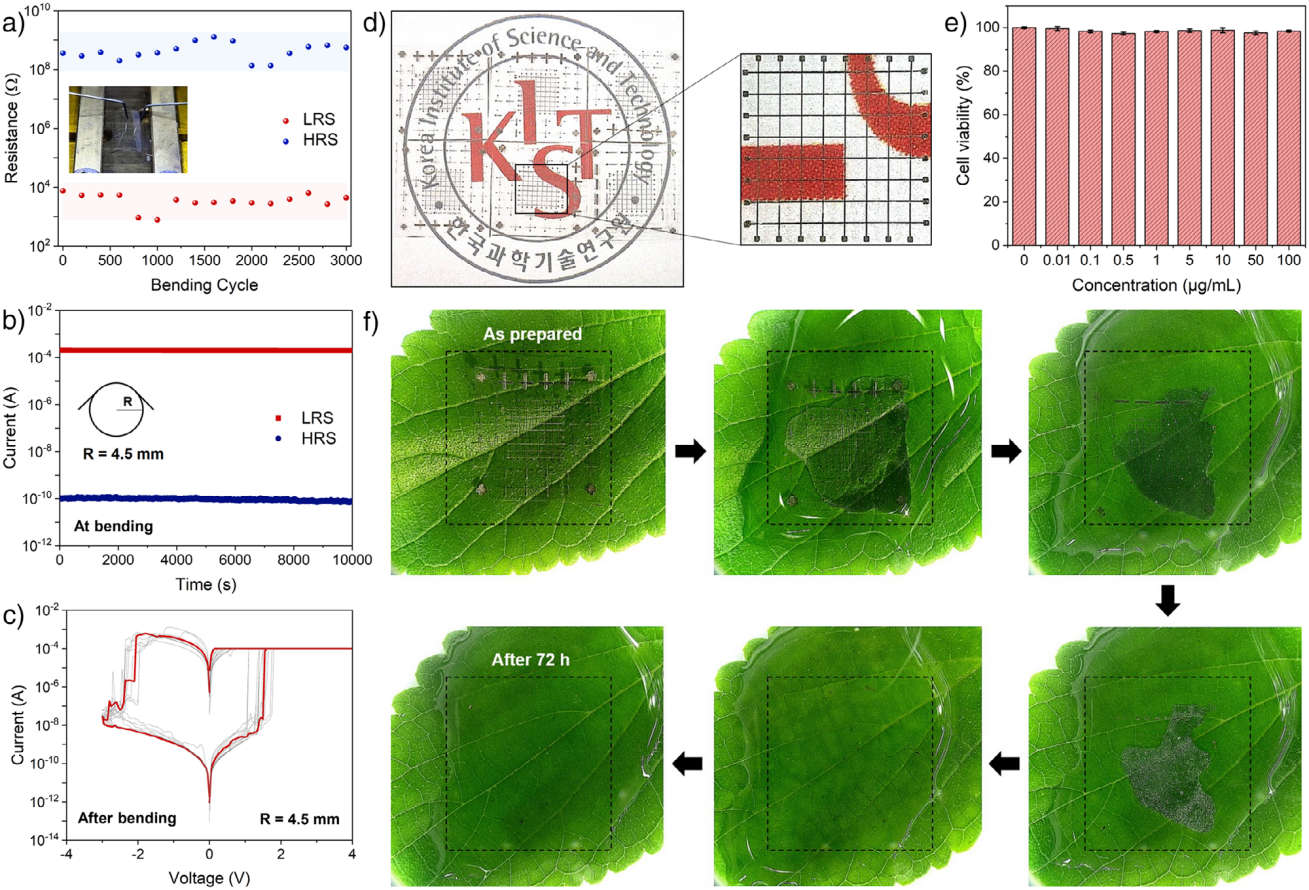
Designer features of PCL‐TEMPO toward soft bioelectronics. a)–c) Flexibility test of PCL‐TEMPO‐based device: (a) Fatigue, (b) performance retention at bending, and c) *I–V* cycles after the fatigue test. d) Photos highlighting the optical transparency of PCL‐TEMPO based device. Inset displays an individual crossbar array with each device in the array assumes an area of 50 x 50 µm^2^. e) Cell viability test on a PCL‐TEMPO thin film. f) On‐demand physical transience test of the device during 72 h of exposure to DI water at room temperature.

The successful application of our polymer in bioelectronics and advanced biorealistic systems hinges on the precise tailoring of its structural and functional properties to meet the demands of practical, real‐world applications. Key attributes include: (i) optical transparency and (ii) biocompatibility, essential for seamless integration with biological tissues and wearable technologies; (iii) mechanical compliance or flexibility, which allows the polymer to withstand various biomechanical stresses encountered during routine activities, and (iv) on‐demand physical transience, which not only enhances the sustainability of the material but also permits the programmed dissociation or degradation of devices post‐use, such as in the case of temporary implants.^[^ ^4^, ^19^ ^]^ Figure 4 illustrates these designer features, of a **PCL‐TEMPO** based crossbar‐type device on a biodegradable substrate, poly(lactic acid) (PLA). The fabricated device assumed identical active layer thickness to the dot arrays (in Figure 3), where molybdenum electrodes were utilized to represent more holistic view on the physical transience of the device (Figure 1). Note that the molybdenum was chosen for its characteristic dissolution in water over extended period time.^[^ ^10^ ^]^ The device was highly thin (∼20 µm) and flexible (Figure 4a–c) and exhibited optical transparency (Figure 4d). Importantly, we found that replacing the electrode and the substrate materials with molybdenum or PLA did not significantly affect the memory performance. The device displayed no obvious cytotoxicity as we conducted a cell viability test to assess its potential for various biointerfaces (Figure 4e). Specifically, the test was performed using mouse normal fibroblast cell lines (L929) to evaluate the toxicity of **PCL‐TEMPO**. A range of sample concentrations (0 to 100 µg mL^−1^) was tested, and no significant cytotoxicity was observed across all concentrations. As briefly mentioned above, the device also displayed retention of its performance on iterative cycles of real‐life mechanical stresses, such as bending. In particular, we observed that the device displayed no significant deterioration in its memory performance over >3000 bending cycles, at a bending radius of 4.5 mm (Figure 4a). Note the device was also stably operable at constant bending strain (Figure 4b) and maintained its stable *I–V* cycles after the fatigue test (Figure 4c). Lastly, the device exhibited substantial on‐demand physical transience, at a very mild environmental condition of applying DI water (Figure 4f). Specifically, the device displayed its disappearance before 72 h of exposure to the water at room temperature. Note that these features are also expected to be adjustable by dedicated device engineering, as with the device metrics such as switching voltage (see above). These designer features of **PCL‐TEMPO** based memory device are expected to contribute significantly to the practical implementation in biomedical and related areas of applications.

Given the unique design features of **PCL‐TEMPO** based devices, further consideration of their practical applications is worthwhile. While the physical transience of these devices in physiological environments is highly advantageous for implantable neuromorphic systems (for example, by eliminating the need for post‐surgical removal), it may not be suitable for long‐term implantation or wearable applications that require delayed or no degradation under physiological conditions. To address these concerns, we demonstrate that appropriate packaging or passivation strategies can significantly extend the functional lifespan of our system. Specifically, we show that passivation with PCL and poly(dimethylsiloxane) (PDMS) can either (i) substantially delay or (ii) completely prevent device dissociation in physiological environments (Figure S15; see details in the Experimental Section). An important point to clarify is the origin of the physical transience observed in our device system. We find that it is more accurately described as the physical dissociation of **PCL‐TEMPO** rather than its chemical degradation into monomeric units. However, prolonged exposure of the dissociated polymer chains in physiologically relevant environments would eventually lead to chemical degradation, justifying the concept depicted in Figure 1.^[^ ^60^ ^]^ Furthermore, the increased surface area of the thin‐film **PCL‐TEMPO** and the dissolution of the electrode may also accelerate this process. To support this claim, we monitored the dissolution of **PCL‐TEMPO** in water using ^1^H NMR analysis over time (Figure S16). Notably, this distinction in the origin of physical transience does not affect the classification of our device as physically transient, in line with transient memristors reported elsewhere.^[^ ^10^ ^]^ In practical applications, there may be scenarios where rapid chemical degradation of the device is required. To explore this, we conducted an accelerated chemical degradation test under relatively mild conditions of an acid or a base at elevated temperature (Figure S17). Under these conditions, **PCL‐TEMPO** fully degraded into water‐soluble monomers of PCL and hydroxylated TEMPO (Figure 1). Another key consideration is the potential cytotoxicity of TEMPO during device degradation. However, an approximate calculation indicates that the total **PCL‐TEMPO** content in a single crossbar device is only ∼5 µg, which is significantly below established cytotoxicity thresholds (Figure 4e). Notably, the active layer comprises only a small mass fraction (< 0.5%) of the entire device, further minimizing any potential toxicity concerns. Lastly, assessing potential performance degradation due to humidity is crucial for the practical operation of **PCL‐TEMPO** based memristive devices as the molecular structure of **PCL‐TEMPO** could facilitate ion solubilization and partial dissolution under high relative humidity (RH).^[^ ^24^ ^]^ To investigate this, we evaluated the retention characteristics of the device at various humidity levels (20%, 40%, 60%, 80%, and 90%) (Figure S18). While a minor change in the HRS current was observed, consistent with our previous work,^[^ ^24^ ^]^ no significant performance deterioration was detected across all tested humidity levels. Furthermore, we found that the passivation strategy discussed earlier effectively mitigates humidity‐induced variations, with PDMS passivation significantly reducing HRS current fluctuations, even at 90% relative humidity (Figure S19).

Overall, to emphasize the exceptional memory performance and on‐demand degradability of the **PCL‐TEMPO** based device, we directly compared its metrics with other classes of physically transient memristive devices (Table 1).^[^ ^10^ ^]^ Compared to reported organic memristive devices, our device demonstrated exceptional performance, including no need for a forming process, low operating voltage, a large resistance window, high endurance cycles, long retention time, and no need for specific chemical agents for the physical transience. Also, while not explicitly addressed in Table 1, we summarize the key advantages of this work over our previous study on the prototype radical polymer. First, this study presents the first explicit demonstration of physical transience and mechanical compliance in radical polymer‐based memristors, confirming stable memristive performance across various substrates and device configurations. Second, it achieves a notable improvement in device yield, partially addressing a longstanding challenge in organic memristors and representing a significant step toward their practical implementation. Third, this work indicates the versatility and generalizability of our molecular engineering strategy, which can be extended to a broader class of synthetic and biopolymers with an expanded functional window (e.g., self‐healing and light responsiveness). Notably, we are currently developing various multifunctional memristive devices based on polycarbonates and polypeptides, which will be discussed in detail in our forthcoming studies.

**Table 1:** Comparison of device performance metrics with previous literature (Reproduced from ^[^ ^10^ ^]^ with permission from the Royal Society of Chemistry).^[^ ^10^ ^]^

Active layer	Forming process	On/off Ratio	Cycle	Retention time (s)	Dissociation/degradation method	Flexibility (bending radius)	Switching mechanism	Ref.
Silk fibroin	Required	>10^5^	100	>8 × 10^4^	Buffer solution	Down to 10 mm	Extrinsic	[21]
Silk fibroin	Required	>10^2^	30	>3 × 10^5^	DI water	Qualitative	Extrinsic	[49]
Silk fibroin	Required	>10^2^	50	>10^4^	DI water	–	Extrinsic	[50]
Egg albumen	–	>10^2^	120	>10^4^	DI water	–	Extrinsic	[51]
Keratin	Required	>10^3^	100	>10^4^	DI water	–	Extrinsic	[22]
Gelatin	Not required	∼10^2^	100	>10^4^	DI water	Down to 10 mm	Extrinsic	[52]
Soya protein	–	∼10^5^	–	>10^5^	DI water	Qualitative	Intrinsic	[53]
Glucose	Required	>10^3^	100	>10^4^	DI water	Down to 15 mm	Extrinsic	[54]
Glucose	–	∼10	100	>10^4^	DI water	–	Extrinsic	[55]
Chitosan	Required	>100	60	>10^4^	DI water	Down to 5 mm	Extrinsic	[23]
Cellulose	Required	>10^7^	20	>10^4^	Natural soil	–	Extrinsic	[56]
Pectin	Not required	∼25	500	>10^4^	DI water	Down to 5 mm	Extrinsic	[57]
Lactose	–	>10	100	>5 × 10^3^	DI water	–	Extrinsic	[58]
PVP	–	>10^5^	–	>10^4^	DI water	Down to 10 mm	Intrinsic	[59]
PCL‐TEMPO (This work)	Not required	>10^6^	>250 (DC)	>10^4^	DI water	Down to 4.5 mm	Intrinsic	

## Conclusion

In this work, we presented a nonconjugated polymer derivative with intrinsic memristivity, which enabled high‐performance soft memory with on‐demand physical transience. The inherent memory function of the base material resulted in an exceptional resistance window, superior endurance cycles, and extended retention times for an organic memristive material. A biocompatible variant of the prototype device exhibited complete dissociation under mild environmental conditions, while maintaining excellent performance compared to both dissociable and nondissociable organic memristive devices. This work represents the first instance of unconventional soft materials integrated into transient memristive devices, highlighting their potential to be readily adapted for future applications in soft bioelectronics and biorealistic systems.

## Supporting Information

The authors have cited additional references within the Supporting Information.^[61–64]^

## Conflict of Interests

The authors declare no conflict of interest.

## Supporting information



Supporting Information

## Data Availability

The data that support the findings of this study are available from the corresponding author upon reasonable request.

## References

[1] W. Wondrak , Microelectron. Reliab. 1999, 39, 1113–1120.

[2] M. G. Pecht , F. R. Nash , Proc. IEEE 1994, 82, 992–1004.

[3] A. Hanif , Y. Yu , D. DeVoto , F. Khan , IEEE Trans. Power Electron. 2019, 34, 4729–4746.

[4] S.‐K. Kang , L. Yin , C. Bettinger , MRS Bull. 2020, 45, 87–95.

[5] S.‐W. Hwang , H. Tao , D.‐H. Kim , H. Cheng , J.‐K. Song , E. Rill , M. A. Brenckle , B. Panilaitis , S. M. Won , Y.‐S. Kim , Y. M. Song , K. J. Yu , A. Ameen , R. Li , Y. Su , M. Yang , D. L. Kaplan , M. R. Zakin , M. J. Slepian , Y. Huang , F. G. Omenetto , J. A. Rogers , Science 2012, 337, 1640–1644. 23019646 10.1126/science.1226325 PMC3786576

[6] W. B. Han , J. H. Lee , J.‐W. Shin , S.‐W. Hwang , Adv. Mater. 2020, 32, 2002211. 10.1002/adma.202002211 32974973

[7] M. Shahabuddin , M. N. Uddin , J. I. Chowdhury , S. F. Ahmed , M. N. Uddin , M. Mofijur , M. A. Uddin , Int. J. Environ. Sci. Technol. 2023, 20, 4513–4520.

[8] K. Grant , F. C. Goldizen , P. D. Sly , M.‐N. Brune , M. Neira , M. van den Berg , R. E. Norman , Lancet Glob. Health 2013, 1, e350–e361. 25104600 10.1016/S2214-109X(13)70101-3

[9] I. Tzouvadaki , P. Gkoupidenis , S. Vassanelli , S. Wang , T. Prodromakis , Adv. Mater. 2023, 35, 2210035. 10.1002/adma.202210035 36829290

[10] W. Hu , B. Yang , Y. Zhang , Y. She , J. Mater. Chem. C 2020, 8, 14695–14710.

[11] K. Zheng , F. Gu , H. Wei , L. Zhang , X. a. Chen , H. Jin , S. Pan , Y. Chen , S. Wang , Small Methods 2023, 7, 2201534. 10.1002/smtd.202201534 36813751

[12] M. B. Durukan , M. O. Cicek , D. Doganay , M. C. Gorur , S. Çınar , H. E. Unalan , Adv. Funct. Mater. 2022, 32, 2106066.

[13] Y. Guo , M. Zhong , Z. Fang , P. Wan , G. Yu , Nano Lett. 2019, 19, 1143–1150. 30657695 10.1021/acs.nanolett.8b04514

[14] M. Hao , L. Li , S. Wang , F. Sun , Y. Bai , Z. Cao , C. Qu , T. Zhang , Microsyst. Nanoeng. 2019, 5, 9. 31057936 10.1038/s41378-019-0047-4 PMC6409363

[15] P. Yao , H. Wu , B. Gao , J. Tang , Q. Zhang , W. Zhang , J. J. Yang , H. Qian , Nature 2020, 577, 641–646. 31996818 10.1038/s41586-020-1942-4

[16] J. J. Yang , D. B. Strukov , D. R. Stewart , Nat. Nanotechnol. 2013, 8, 13–24. 23269430 10.1038/nnano.2012.240

[17] L. Yuan , S. Liu , W. Chen , F. Fan , G. Liu , Adv. Electron. Mater. 2021, 7, 2100432.

[18] S. Gao , X. Yi , J. Shang , G. Liu , R.‐W. Li , Chem. Soc. Rev. 2019, 48, 1531–1565. 30398508 10.1039/c8cs00614h

[19] A. Fanelli , D. Ghezzi , Curr. Opin. Biotechnol. 2021, 72, 22–28. 34464936 10.1016/j.copbio.2021.08.011

[20] C. Hu , L. Wang , S. Liu , X. Sheng , L. Yin , ACS Nano 2024, 18, 3969–3995. 38271679 10.1021/acsnano.3c11832

[21] X. Ji , L. Song , S. Zhong , Y. Jiang , K. G. Lim , C. Wang , R. Zhao , J. Phys. Chem. C 2018, 122, 16909–16915.

[22] Q. Lin , S. Hao , W. Hu , M. Wang , Z. Zang , L. Zhu , J. Du , X. Tang , J. Mater. Chem. C 2019, 7, 3315–3321.

[23] N. R. Hosseini , J.‐S. Lee , Adv. Funct. Mater. 2015, 25, 5586–5592.

[24] J. Ko , D. Kim , Q. H. Nguyen , C. Lee , N. Kim , H. Lee , J. Eo , J. E. Kwon , S.‐Y. Jeon , B. C. Jang , S. G. Im , Y. Joo , Sci. Adv. 2024, 10, eadp0778. 39121228 10.1126/sciadv.adp0778 PMC11313951

[25] Y. Joo , V. Agarkar , S. H. Sung , B. M. Savoie , B. W. Boudouris , Science 2018, 359, 1391–1395. 29567710 10.1126/science.aao7287

[26] I. Yu , D. Jeon , B. Boudouris , Y. Joo , Macromolecules 2020, 53, 4435–4441.

[27] Q. V. Thi , Q. H. Nguyen , Y.‐S. Choi , S.‐Y. Jeon , B. W. Boudouris , Y. Joo , JACS Au 2024, 4, 690–696. 38425938 10.1021/jacsau.3c00743 PMC10900204

[28] J. Ko , Q. H. Nguyen , Q. V. Thi , Y. Joo , Chem. Phys. Rev. 2023, 4, 041310.

[29] T. P. Nguyen , A. D. Easley , N. Kang , S. Khan , S. M. Lim , Y. H. Rezenom , S. Wang , D. K. Tran , J. Fan , R. A. Letteri , X. He , L. Su , C. H. Yu , J. L. Lutkenhaus , K. L. Wooley , Nature 2021, 593, 61–66. 33953410 10.1038/s41586-021-03399-1

[30] Y. Xu , K. Zhang , S. Reghu , Y. Lin , M. B. Chan‐Park , X. W. Liu , Biomacromolecules 2019, 20, 949–958. 30629424 10.1021/acs.biomac.8b01577

[31] Y. Dai , Z. Hu , X. Wang , X. Liu , Y. Li , Y. Shi , Y. Chen , Polym. Chem. 2021, 12, 2592–2597.

[32] D. F. Shellhamer , Z. J. Beavis , D. L. Brady , M. S. Bucardo , S. L. Elwin , N. Fiorella , L. Gomez , S. Van Horne , M. C. Perry , Results Chem. 2020, 2, 100015.

[33] L. Rostro , S. H. Wong , B. W. Boudouris , Macromolecules 2014, 47, 3713–3719.

[34] J. Tan , C. Li , K. De Bruycker , G. Zhang , J. Gu , Q. Zhang , RSC Adv. 2017, 7, 51763–51772.

[35] D. M. Walters , R. Richert , M. D. Ediger , J. Chem. Phys. 2015, 142, 134504. 25854250 10.1063/1.4916649

[36] S. J. Blanksby , G. B. Ellison , Acc. Chem. Res. 2003, 36, 255–263. 12693923 10.1021/ar020230d

[37] M. Labet , W. Thielemans , Chem. Soc. Rev. 2009, 38, 3484. 20449064 10.1039/b820162p

[38] M. A. Woodruff , D. W. Hutmacher , Prog. Polym. Sci. 2010, 35, 1217–1256.

[39] C. Baptista , A. Azagury , H. Shin , C. M. Baker , E. Ly , R. Lee , E. Mathiowitz , Polymer 2020, 191, 122227.

[40] M. K. Singh , M. Hu , Y. Cang , H. P. Hsu , H. Therien‐Aubin , K. Koynov , G. Fytas , K. Landfester , K. Kremer , Macromolecules 2020, 53, 7312–7321. 32921812 10.1021/acs.macromol.0c00550 PMC7482400

[41] E. s. M. Yazdani‐pedram , L. H. Tagle , F. R. Diaz , L. Gargallio , D. Radic , Thermochim. Acta 1986, 105, 149–160.

[42] P. C. S. Montserrate , Polym. Bull. 1984, 12, 1738–1744.

[43] W. Brostow , R. Chiu , I. M. Kalogeras , A. Vassilikou‐Dova , Mater. Lett. 2008, 62, 3152–3155.

[44] L. Gao , J. Oh , Y. Tu , T. Chang , C. Y. Li , Polymer 2019, 170, 198–203.

[45] H. A. Lopez‐Pena , L. S. Hernandez‐Munoz , B. A. Frontana‐Uribe , F. J. Gonzalez , I. Gonzalez , C. Frontana , J. Cardoso , J. Phys. Chem. B 2012, 116, 5542–5550. 22510068 10.1021/jp301207v

[46] A. Rose , Phys. Rev. 1955, 97, 1538–1544.

[47] B. C. Jang , H. Seong , S. K. Kim , J. Y. Kim , B. J. Koo , J. Choi , S. Y. Yang , S. G. Im , S.‐Y. Choi , ACS Appl. Mater. Interfaces 2016, 8, 12951–12958. 27142537 10.1021/acsami.6b01937

[48] J. B. Gerken , S. S. Stahl , ACS Cent. Sci. 2015, 1, 234–243. 27162977 10.1021/acscentsci.5b00163 PMC4827547

[49] J. Yong , B. Hassan , Y. Liang , K. Ganesan , R. Rajasekharan , R. Evans , G. Egan , O. Kavehei , J. Li , G. Chana , B. Nasr , E. Skafidas , Sci. Rep. 2017, 7, 14731. 29116250 10.1038/s41598-017-15395-5 PMC5676789

[50] H. Wang , B. Zhu , X. Ma , Y. Hao , X. Chen , Small 2016, 12, 2715–2719. 27028213 10.1002/smll.201502906

[51] X. He , J. Zhang , W. Wang , W. Xuan , X. Wang , Q. Zhang , C. G. Smith , J. Luo , ACS Appl. Mater. Interfaces 2016, 8, 10954–10960. 27052437 10.1021/acsami.5b10414

[52] S. Liu , S. Dong , X. Wang , L. Shi , H. Xu , S. Huang , J. Luo , Nanotechnology 2020, 31, 255204. 32101798 10.1088/1361-6528/ab7a2c

[53] Y. Sun , D. Wen , Y. Xie , F. Sun , X. Mo , J. Zhu , H. Sun , J. Phys. Chem. Lett. 2019, 10, 7745–7752. 31773960 10.1021/acs.jpclett.9b03238

[54] S. P. Park , Y. J. Tak , H. J. Kim , J. H. Lee , H. Yoo , H. J. Kim , Adv. Mater. 2018, 30, 1800722. 10.1002/adma.201800722 29761552

[55] S. P. Park , H. J. Kim , J. H. Lee , H. J. Kim , J. Inf. Disp. 2019, 20, 231–237.

[56] U. Celano , K. Nagashima , H. Koga , M. Nogi , F. Zhuge , G. Meng , Y. He , J. De Boeck , M. Jurczak , W. Vandervorst , T. Yanagida , NPG Asia Mater 2016, 8, e310.

[57] J. Xu , X. Zhao , Z. Wang , H. Xu , J. Hu , J. Ma , Y. Liu , Small 2019, 15, 1803970. 10.1002/smll.201803970 30500108

[58] Y. Guo , W. Hu , F. Zeng , C. Zhang , Y. Peng , Y. Guo , Org. Electron. 2020, 83, 105750.

[59] Z. Zhou , H. Mao , X. Wang , T. Sun , Q. Chang , Y. Chen , F. Xiu , Z. Liu , J. Liu , W. Huang , Nanoscale 2018, 10, 14824–14829. 30043803 10.1039/c8nr04041a

[60] H. Sun , L. Mei , C. Song , X. Cui , P. Wang , Biomaterials 2006, 27, 1735–1740. 16198413 10.1016/j.biomaterials.2005.09.019

