# Supplementary material for: A Biodegradable Radical Polymer Enables High‐Performance, Physically Transient Organic Memory
Source: Angew Chem Int Ed Engl. 2025 May 12;64(27):e202422826. doi: 10.1002/anie.202422826 (PMC12207568; doi:10.1002/anie.202422826)
Supplement: Supplementary file 1 — Supporting Information [file ANIE-64-e202422826-s001.docx]

Supporting Information

A Biodegradable Radical Polymer Enables High-Performance, Physically Transient Organic Memory

Jaehyoung Ko,^†^ Soeun Kim,^†^ Daeun Kim, Taeho Lim, Soyeoung Jin, Youngdo Jeong, Yongho Joo,* Sangho Cho*

† These authors contributed equally to this work.

**Contents**

Experimental Section

Synthesis of PCL-TEMPO

Scheme S1

Figure S1 – Figure S19

Table S1

References

**Experimental Section**

*Materials*: All chemicals were purchased from commercial sources and used without further purification unless otherwise noted. These include polycaprolactone (PCL, Sigma-Aldrich), polyethylene terephthalate (PET, Youlchon Chemical, South Korea), poly(lactic acid) (PLA, Youlchon Chemical, South Korea), 4-hydroxy-2,2,6,6-tetramethylpiperidine 1-oxyl (TEMPOL, > 98%, Thermo Fisher Scientific), iron (Ⅱ) sulfate heptahydrate (FeSO_4_•7H_2_O, >99%, Thermo Fisher Scientific), hydrogen peroxide solution (30% in H_2_O, Sigma Aldrich), cyclohexanone (99%, Daejung), *N*-bromosuccimide (NBS, >98%, TCI), *p*-toluenesulfonic acid monohydrate (*p*TSA, >98.5%, Alfa Aesar), 3-chloroperbenzoic acid (*m*CPBA, <77%, Sigma-Aldrich), benzyl alcohol (99.5%, Alfa Aesar), tin (Ⅱ) 2-ethylhexanoate (Sn(Oct)_2_, 95%, Sigma-Aldrich), anhydrous dichloromethane (DCM, Daejung), anhydrous dimethylformamide (DMF, Daejung), 3,3′-dithiodipropionic acid (99%, Sigma-Aldrich), *N,N′*-dicyclohexylcarbodiimide (DCC, 98%, Daejung), 4-(dimethylamino)pyridine (DMAP, 99%, Daejung), triethylamine (Et_3_N, Daejung), DL-dithiothreitol (DTT, 99%, Daejung), and dimethylsulfoxide (DMSO, 99.5%, Daejung). Note the benzyl alcohol was dried over CaH_2_ and distilled before use.

*Sample preparation*: For the device fabrication, designated amounts of **PCL-TEMPO** were dissolved in DCM or DMF, and then filtered twice with a syringe filter (0.1 μm, 13 mm, Whatman). Electrochemical measurements were carried out for 0.1 mM acetonitrile solution of **PCL-TEMPO**, with 0.1 M tetrabutylammonium hexafluorophosphate as an electrolyte. For ESR measurements, **PCL-TEMPO** was dissolved in methylene chloride to give a molar concentration of 0.1 mM. A commercial ESR tube container with an effective sample volume of 130 μL was utilized. For the flexibility test, a crossbar device based on **PCL-TEMPO** was fixed with a dedicated mechanical jig. The bending radius was measured by image analysis of the bent film at the jig, and the bending and relaxation were applied to the film through a mechanical dial attached to the jig, with 50 mm^-1^ resolution of precision. For the on-demand degradability test, the device with PLA substrate was placed on a leaf. We then added a designated amount of DI water to the device, so that the entirety of the device is fully wet and submerged for the duration of 72 h. To minimize the evaporation of the DI water, the system was properly sealed with a transparent plastic lid. For water dissolution test,

*Device fabrication*: For a general process of device fabrication, a 1.5 cm × 1.5 cm of thermal oxide (oxide thickness, 300 Å), PET, or PLA substrates were thoroughly rinsed. For the rinsing, the thermal oxide was treated with a standard protocol using acetone, isopropyl alcohol, and DI water using bath sonication (JAC-3010, Kodo, Japan). In the case of PET and PLA substrates, the protocol without acetone was applied. The active layers of **PCL-TEMPO** were spun coat (Won corporation, South Korea) to achieve the designated layer thicknesses. For all the experiments unless otherwise noted, 100 nm of active layer thickness was applied. For control experiments, we fabricated devices following similar protocol with 50 nm of active layer thickness. The electrodes were deposited by e-beam evaporation of gold, copper, or molybdenum sources, to the thicknesses of 30 nm for the bottom electrodes, and 50 nm for the top electrodes using dedicated shadow masks (dot and crossbar arrays).

*Characterization*: The chemical structure and molar ratio of each monomer were observed by ^1^H and ^13^C NMR (Bruker, Avance 400 MHz) spectroscopy using chloroform-*d* (CDCl_3_). Chemical shifts were referenced to solvent resonance signals. The synthesized polymers’ *M*_n_ and *Ð* were measured by gel permeation chromatography (GPC, JASCO PU-2080 plus) equipped with a refractive detector (RI-2931) and a UV detector (UV-2075, 254 nm). THF was used for the mobile phase at 40 °C with 1 mL min^−1^ flow rate. The molecular weight was calibrated based on polystyrene (PS) standards. FTIR spectra were recorded from the attenuated total reflectance (ATR) mode on powder samples. Thermal gravimetric analysis (TGA) was performed using a TA Instrument TGA Q50-1200 (heating rate, 10 °C min^−1^, at inert atmosphere). Differential scanning calorimetry (DSC) was performed using a TA Instrument DSC Q20-1426 (cooling/heating rate, 10 °C min^−1^, at inert atmosphere). For the electrical measurements, a multipurpose parameter analyzer (Keithley-4200 SCS, Tektronix, United States) equipped with preamplifier (4225-RPM, Tektronix, United States) was utilized. A dedicated probe tip (tip diameter of 0.4 μm, Modusys, South Korea) was utilized for the analysis of the dot arrays. Electrochemical analysis was carried out on an electrochemical workstation (Gamry Instruments, United States). The voltammogram was obtained at a scan rate of 10 mV s^-1^. ESR measurements were carried out with an EPR spectrometer (JES-FA100, JEOL). All the measurements were carried out at room temperature. A solution of 4-hydroxy TEMPO was used as an external reference. Experimental parameters are as follows: microwave frequency, 9.226 GHz; microwave power, 0.998 mW; modulation amplitude, 1 mT; modulation frequency, 100 kHz; time constant, 0.03 s. For the temperature-dependent measurements, a hot chuck temperature controller (MS Tech, South Korea) was utilized, where temperatures were digitally controlled by inbuilt feedback control. To assess **PCL-TEMPO** dissolution in water at ambient conditions, ^1^H NMR was performed over time (1 day to 4 weeks). For the humidity-dependent device performance measurement, a multipurpose gas sensing measurement system (Phocos, South Korea) was used. Varying relative humidity (RH) sweep was carried out on a programmed input of either an argon gas or a water vapor, where the maximum flow rate was fixed at 500 standard cubic centimeter per minute. All the electrical measurements at variable RH were carried out in a dedicated sealed chamber. For every measurement, at least 1 hour of equilibration step before the actual measurement was taken.

*Cytotoxicity of PCL-TEMPO*: L929 cells (NCTC clone 929: CCL 1, American Type Culture Collection [ATCC], Manassas, VA, USA: ECACC No. 88102702, European Collection of Cell cultures, Salisbury, Wiltshire SP4 0JG, UK) were cultured in Dulbecco’s Modified Eagle Medium (DMEM; Welgene), supplemented with 10% fetal bovine serum (FBS; Sigma Aldrich) and 1% Zellshiled (Minerva Biolabs). The CellTiter 96 Aqueous One Solution Cell Proliferation Assay (Promega) was used for cell viability study. Briefly, cells were seeded in a sterile 96-well plate (SPL life sciences) at a density of 5×10^4^ cells per well and allowed to settle for 24 h with incubation at 37 °C in a 5 % CO_2_. The **PCL-TEMPO** was dissolved in media at concentrations ranging from 0.01 to 100 μg mL^-1^. The cell media were replaced with the sample solutions and incubated for another 24 h under incubation at 37 °C and 5 % CO_2_. Afterward, the assay reagent was treated to each well, mixed with the medium at a 9:1 ratio, and incubated for an additional 2 h. The absorbance was recorded with a plate reader (SpectraMax) at 490 nm, with each data point measured in triplicate.

*Degradability of PCL-TEMPO*: **PCL-TEMPO** was hydrolyzed under accelerated acid and base conditions to identify degradation products. For acid hydrolysis, **PCL-TEMPO** powder (30 mg) was dispersed in 1 M HCl/ D_2_O (5 mL) and heated at 80 ºC for 24 h. The solvent was removed under vacuum, and the residue was dissolved in DMSO-*d*_6_ (~1 mL), then filtered (0.45μm) for ^1^H NMR analysis. Base hydrolysis followed the same procedure at 90 ºC for 24 h, with 1 M HCl added to adjust pH.

*Dissolution of PCL-TEMPO in water:* To assess **PCL-TEMPO** dissolution in water at ambient conditions, ^1^H NMR was performed over time. **PCL-TEMPO** (60 mg) was dispersed in D_2_O (9 mL) at 40 ~ 45 ºC. At each interval, 0.5 mL was sampled and filtered (0.45μm).

*Passivation of PCL-TEMPO based devices*: For the delayed transience, thin PCL layer was spun coat on the **PCL-TEMPO** based device. Briefly, PCL was thermally dissolved in acetonitrile, which was then spun coat to give a PCL layer of thickness approximately 100 nm. For the complete passivation, a silicone elastomer base and a curing agent (Sylgard 184, Dow) were mixed in 10:1 weight ratio. The mixture was placed in a petri dish to give a thin layer, which was evacuated briefly to remove the air bubbles inside the mixture. PCL-TEMPO based devices were then immersed into the mixture, which was heated at 90 ˚C for 3 h on a hot plate. The resultant device was cut into square to represent the passivated device.

**Synthesis of PCL-TEMPO**


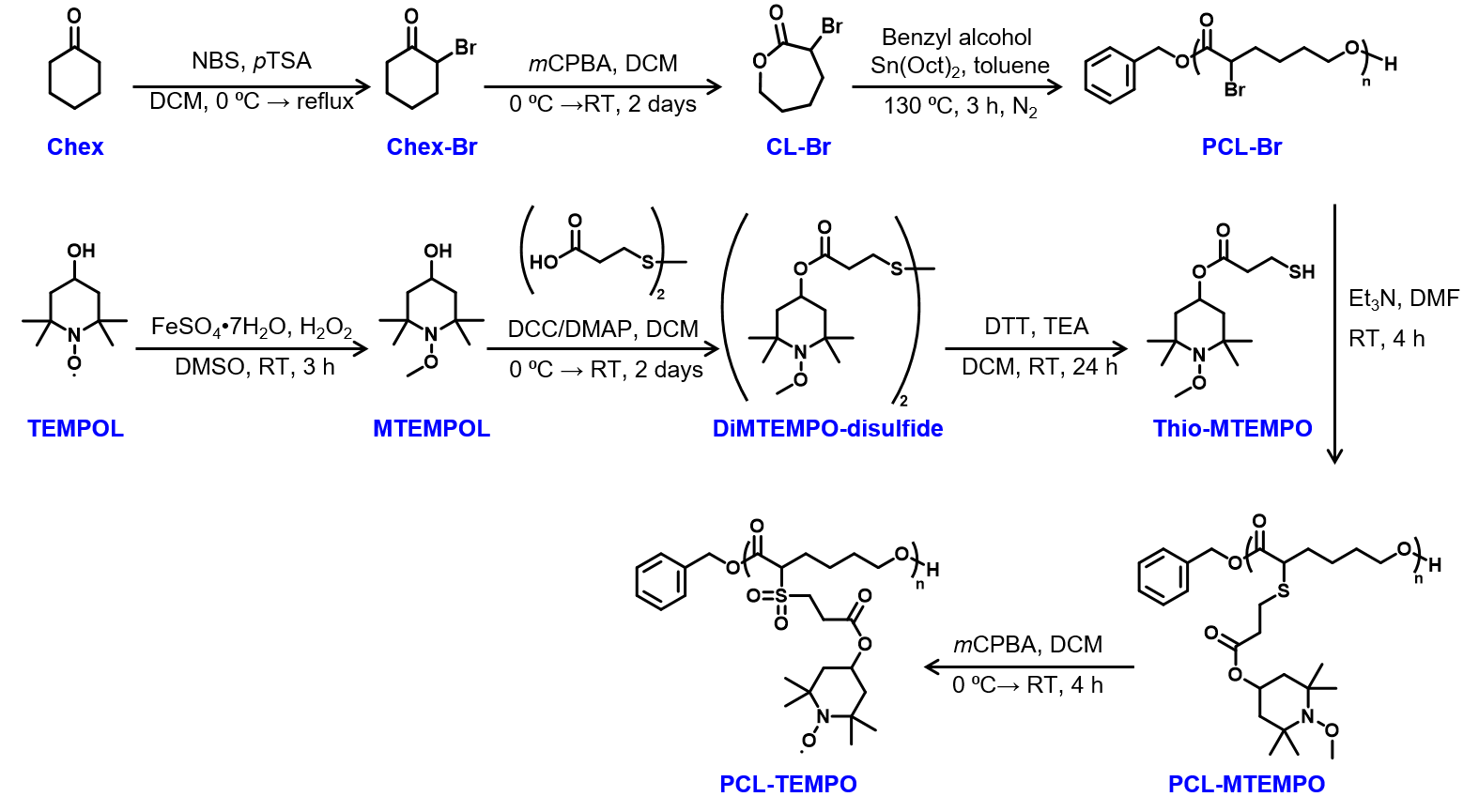


Scheme S1. Synthesis of PCL-TEMPO

*1. Synthesis of 1-Methoxy-2,2,6,6-tetramethylpiperidine-4-ol (MTEMPOL) via methylation of TEMPOL*

In a 2-neck RBF (250 mL), 4-hydroxyl TEMPO (9.2 g, 53.41 mmol, 1 equiv.) and FeSO_4_•7H_2_O (17.82 g, 64.09 mmol, 1.2 equiv.) were dissolved in DMSO (200 mL). 30 wt% aq. H_2_O_2_ solution (2.76 g, 81.19 mmol, 1.52 equiv.) was slowly added into the reaction flask over 30 min. After 2.5 h, reaction solution was moved to the Erlenmeyer flask (500 mL) and cooled to 0 ºC using an ice bath. 150 mL of water was slowly added into the flask and subsequently aqueous NaOH solution (70 mL, 2.7 mm) was slowly added. After stirring for 1 h, the reaction solution was extracted with diethyl ether (200 mL) three times. The organic layer was further washed with brine and collected. After drying it over MgSO_4_, it was filtered. The filtrate was concentrated and further purified by column chromatography (EA:Hex = 1:9, 2:8, v/v). It was obtained as white powder (5.55 g, 55.5%). ^1^H NMR (400 MHz, CDCl_3_, ppm) δ 3.94 (m, 1H), 3.61(s, 3H), 1.78 (d, 2H), 1.46 (t, 2H), 1.21 (s, 6H), 1.13 (s, 6H). ^13^C NMR (101 MHz, CDCl_3_) δ 65.60, 63.46, 60.13, 48.47, 33.25, 21.00.

*2. Synthesis of DiMTEMPO-disulfide via DCC/DMAP coupling reaction*

In a one-neck RBF (250 mL), 3,3′-dithiodipropionic acid (2.47 g, 11.75 mmol, 1 equiv.), MTEMPOL (5.5 g, 29.37 mmol, 2.5 equiv.), and DMAP (0.287 g, 2.35 mmol, 0.2 equiv.) were dissolved in DCM (100 mL). After cooled to 0 ºC using an ice bath, DCC (5.65 g, 28.37 mmol, 2.33 equiv.) was added. After 1 h, the ice bath was removed and the stirring was kept for 2 days at RT. After diluting with DCM (~ 100 mL), the dicyclohexylurea (DCC, white powder) was filtered. The filtrate was concentrated in vacuum and made as a power via adsorption with silica gel (~30 mL) to be further purified by column chromatography on silica gel with an eluent (EA:Hex = 1:9, v/v). It was obtained as white solid (4.9 g, 76.01%). ^1^H NMR (400 MHz, CDCl_3_, ppm) δ 5.02 (m, 1H), 3.60 (s, 3H), 2.89 (t, 2H), 2.68 (t, 2H), 1.82 (d, 2H), 1.54 (d, 2H), 1.20 (s, 6H), 1.16 (s, 6H). ^13^C NMR (101 MHz, CDCl_3_) δ 171.36, 67.47, 65.63, 60.10, 44.21, 33.36, 33.16, 20.85.

*3. Synthesis of Thio-MTEMPO via reduction of DiMTEMPO-disulfide*

In a one-neck RBF (50 mL), DiMTEMPO disulfide (4.9 g, 8.93 mmol, 1 equiv.) and DTT (2.75 g, 18.86 mmol, 2 equiv.) were dissolved in DCM (30 mL). After N_2_ bubbling for 1 h at RT, Et_3_N (5 mL, 35.71 mmol, 4 equiv.) was added. Stirring was kept for 24 h at RT. The reaction solution diluted with DCM (~ 40 mL) was washed with 1M HCl (20 mL), brine (50 mL) and dried over MgSO_4_. The filtrate was concentrated in vacuum and made as a powder via adsorption with silica gel (~10 mL) to be further purified by flash column chromatography on silica gel with DCM (R_f_ < 0.5). It was obtained as a colorless transparent liquid with stinky smell (2.1 g, 42.7%). ^1^H NMR (400 MHz, CDCl_3_, ppm) δ 5.01 (m, 1H), 3.57 (s, 3H), 2.71 (m, 2H), 2.57 (t, 2H), 1.78 (d, 2H), 1.59 (t, 1H), 1.51 (t, 2H), 1.18 (s, 6H), 1.13 (s, 6H). ^13^C NMR (101 MHz, CDCl_3_) δ 171.23, 67.29, 65.57, 60.05, 44.19, 38.80, 33.11, 20.80, 19.88.

*4. Synthesis of Chex-Br via bromination of cyclohexanone*

In a 1-neck RBF (500 mL), NBS (50.05 g, 281.20 mmol, 1.2 equiv.) and *p*TSA (4.46 g, 23.43 mmol, 0.1 equiv.) were dissolved in DCM (250 mL) at 0 ºC. Separately prepared solution of cyclehexanone (23 g, 234.33 mmol, 1 equiv.) in DCM (70 mL) was added to the flask. The reaction flask was equipped with reflux condenser and refluxed at 40 ºC for 4 h. After cooling, DI-water was added and extracted with DCM. The organic layer was washed with saturated aqueous NaHCO_3_ (100 mL) and brine (100 mL). The organic layer was collected and dried over MgSO_4_, filtered, and concentrated. It was further purified by column chromatography on silica gel with an eluent (EA:Hex = 1:9, v/v). It was obtained as a colorless, transparent liquid (38.4 g, 92.6%). ^1^H NMR (400 MHz, CDCl_3_, ppm) δ 4.43 (t, 1H), 2.97 (m, 1H), 2.31 (m, 2H), 2.22(m, 1H), 2.02 (m, 2H), 1.82-1.74 (m, 2H).

*5. Synthesis of CL-Br via oxidation of Chex-Br*

As one of the reaction intermediates, α-bromo-ε-caprolactone (CL-Br) was synthesized by the classical Baeyer-Viliger oxidation of Chex-Br as reported in previous reports ^[1-4]^ . In a 1-neck RBF (250 mL), α-bromocyclohexanone (Chex-Br, 14g, 79.08 mmol, 1 equiv.) was dissolved in DCM (150 mL) and cooled to 0 ⁰C. *m*-CPBA (16.37 g. 94.89 mmol, 1.2 equiv-real: 23.4 g based on purity) was added to the solution. After stirring in an ice bath for 1 h, the reaction was then stirred for 2 days at RT. Placed in a freezer for 3 h to precipitate 3-chlorobenzoic acid, by product. The solution was then filtered and washed with saturated aqueous Na_2_S_2_O_3_ solution, saturated aqueous NaHCO_3_ solution, and finally with DI-water until neutral pH. The organic layer was dried with MgSO_4_, filtered, and concentrated. The crude product was purified on a silica column using *n*-hexane: EtOAc (9:1, v/v) as an eluent. Slightly brown solid (after cooling) (~6.6 g) was redissovled in diethyl ether (40 mL) at 40 ⁰C and placed in the freezer at −24 ⁰C to obtain pure white crystal as a product (5.02 g, 32.9%). ^1^H NMR (400 MHz, CDCl_3_, ppm) δ 4.84 (t, 1H), 4.70 (m, 1H), 4.29 (m, 1H), 2.12 -1.85 (m, 6H). ^13^C NMR (101 MHz, CDCl_3_) δ 169.72, 69.73, 48.22, 31.78, 29.24, 25.26.

*6. Synthesis of brominated polycaprolactone (PCL-Br)*

In a 1-neck RBF (25 mL), CL-Br (1.96 g, 10.172 mmol, 55 equiv.) was vacuum dried for 1h at 60 ºC. Separately prepared solution of benzyl alcohol (0.02 g, 0.18495 mmol, 1 equiv.) and toluene (10 mL) was treated with N_2_ bubbling. After adding tin catalyst (13 mg, 0.3144 mmol, 0.17 equiv.) into the initiator solution, it was added to the reaction flask and heated at 130 ºC for 3 h. After cooling, the crude was precipitated in cold MeOH (100 ml) on the ice bath three times. By decanting liquid, viscous light brown liquid was obtained after dried in vacuum. (1.84 g, 93%). ^1^H NMR (400 MHz, CDCl_3_, ppm) δ: 7.37 (–C_6_***H***_5_), 5.20 (–C***H***_2_C_6_H_5_), 4.19 (–COC***H***Br– and –CH_2_C***H***_2_O–), 3.60 (–CH_2_C***H***_2_OH), 2.08 (–CHBrC***H***_2_CH_2_–), 1.72-1.48 (–CH_2_C***H***_2_C***H***_2_CH_2_–). ^13^C NMR (101 MHz, CDCl_3_) δ 169.73, 129.17, 128.79, 128.36, 125.43, 65.54, 45.73, 34.39, 27.82, 23.84.

*7. Synthesis of PCL-MTEMPO*

In a RBF (100 mL), PCL-Br (2.83 g, CL-Br; 14.66 mmol) was dissolved in DMF (20 mL). Thio-MTEMPO (4.643 g, 16.86 mmol, 1.15 equiv.) in DMF (10 mL) was added to the flask. After N_2_ bubbling for 10 min, Et_3_N (2.45 mL, 17.59 mmol, 1.2 equiv.) were added. After stirred at RT for 4 h, it was diluted with 75 mL of DCM. The organic layer was washed with saturated aqueous NaHCO_3_ solution and brine. Organic layer was collected, dried over MgSO_4_, and filtered. Filtrate was concentrated and precipitated in cold 90 mL of MeOH two times. After decanting solvent, it was dried via rotary evaporator in vacuum, yielding light brown viscous liquid (4.57 g, 80.22%). ^1^H NMR (400 MHz, CDCl_3_, ppm) 7.37 (–C_6_***H***_5_), 5.20 (–C***H***_2_C_6_H_5_), 5.01 (–CH_2_C***H***CH_2_–), 4.12 (–CH_2_C***H***_2_O–), 3.77 (–CH_2_C***H***_2_OH), 3.59 (–OC***H***_3_), 3.23 (–COC***H***SCH_2_–), 2.85 (–SC***H***_2_CH_2_CO–), 2.55 (–SCH_2_C***H***_2_CO–), 1.90-1.41 (–CHC***H***_2_C***H***_2_C***H***_2_CH_2_O –and –CHC***H***_2_C–), 1.20 (–CC***H***_3_), 1.16 (–CC***H***_3_). ^13^C NMR (101 MHz, CDCl_3_) δ 172.48, 171.25, 67.39, 65.64, 64.98, 60.10, 46.67, 44.22, 34.93, 33.19, 30.95, 28.41, 26.39, 23.81, 20.87.

*8. Synthesis of PCL-TEMPO*

In a one-neck RBF (250 mL), PCL-MTEMPO (4.42 g, CL-MTEMPO; 11.37 mmol) was dissolved in DCM (10 mL). After cooling to 0 ⁰C in an ice bath, the solution of *m-*CPBA (9.52 g) in DCM (100 mL) was slowly added to the reaction flask. After stirring at RT for 4 h, it was washed with cold aqueous media (saturated aqueous NaHCO_3_ solution, brine, and DI-water). The organic layer was dried over MgSO_4_, filtered, and concentrated. It was precipitated in cold diethyl ether and decanted. It was repeated three times by redissolving the precipitate in DCM. To remove the reaction byproducts, *m*CPBA or *m*CBA (*meta*-chlorobenzoic acid) thoroughly, the polymer solution in DCM with 10 wt% concentration was passed through the alumina and concentrated. It was obtained as light orange colored powder (1.6 g, 34.7%). %). ^1^H NMR (400 MHz, CDCl_3_, ppm) 7.37 (–C_6_***H***_5_), 5.20 (–C***H***_2_C_6_H_5_), 5.12 (–CH_2_C***H***CH_2_–), 4.23 (–CH_2_C***H***_2_O–), 3.88 (–CH_2_C***H***_2_OH), 3.61(–COC***H***SO_2_C***H***_2_–), 2.84 (–SC***H***_2_CH_2_CO–), 2.00-1.53 (–CHC***H***_2_C***H***_2_C***H***_2_CH_2_O–and –CHC***H***_2_C–), 1.26 (–CC***H***_3_).


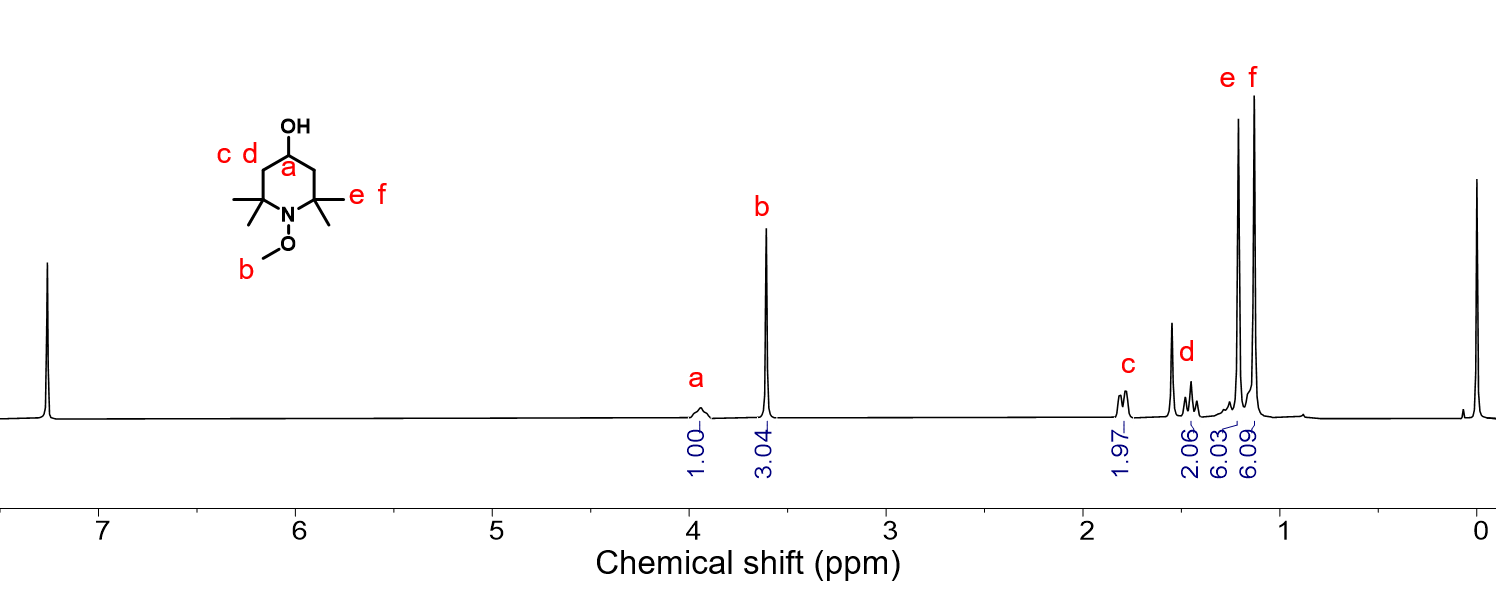


**Figure S1.** ^1^H NMR spectrum of MTEMPOL (400 MHz, CDCl_3_)


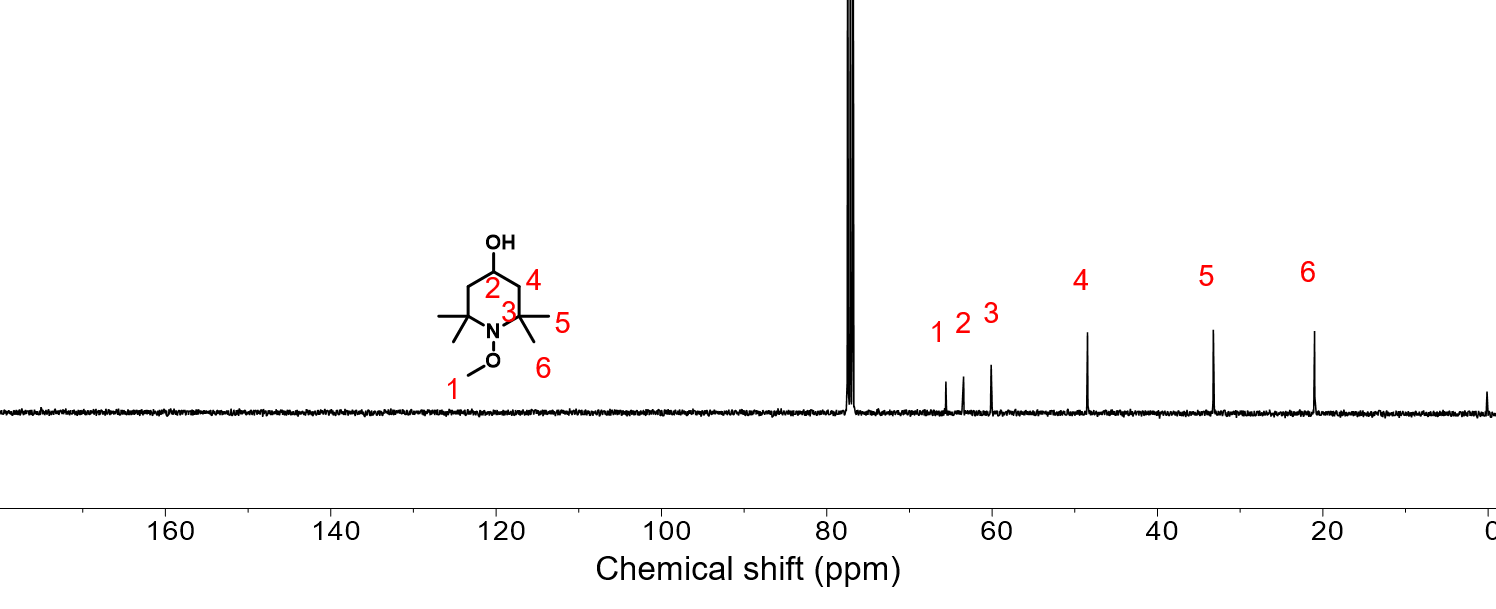


**Figure S2.** ^13^C NMR spectrum of MTEMPOL (101 MHz, CDCl_3_)


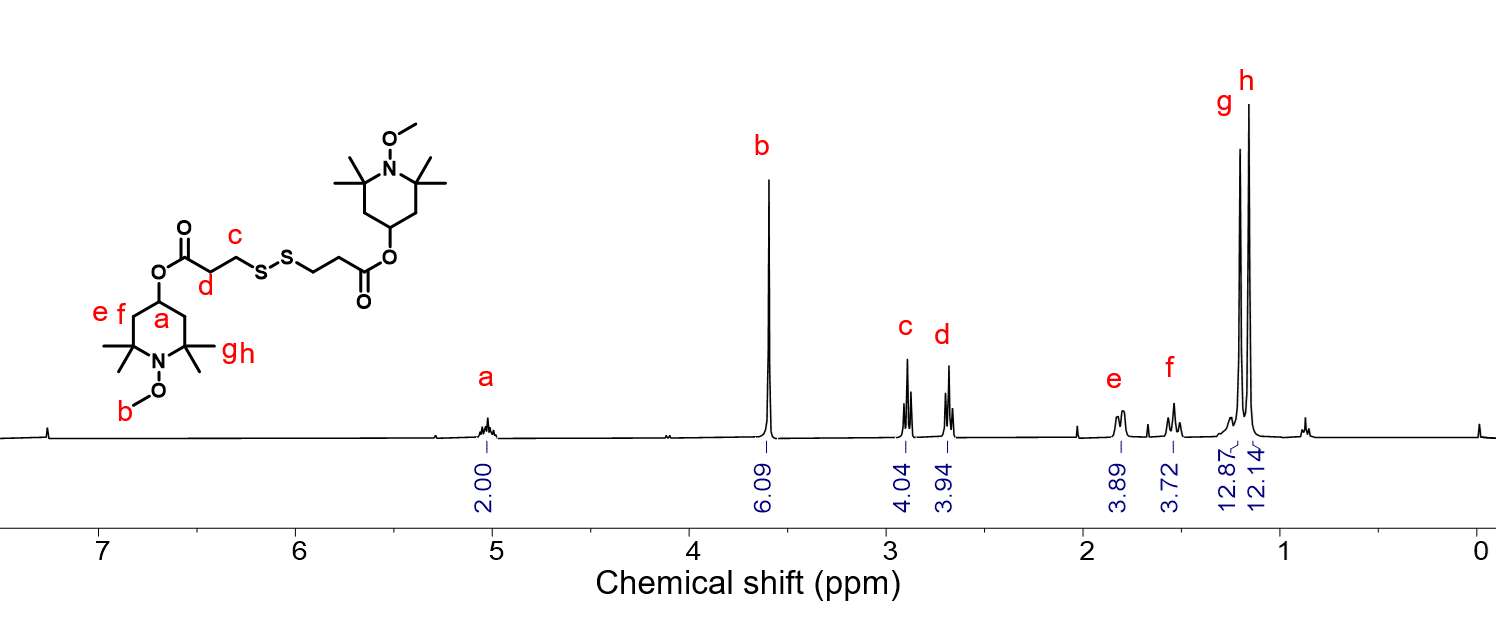


**Figure S3**. ^1^H NMR spectrum of DiMTEMPO-disulfide (400 MHz, CDCl_3_)


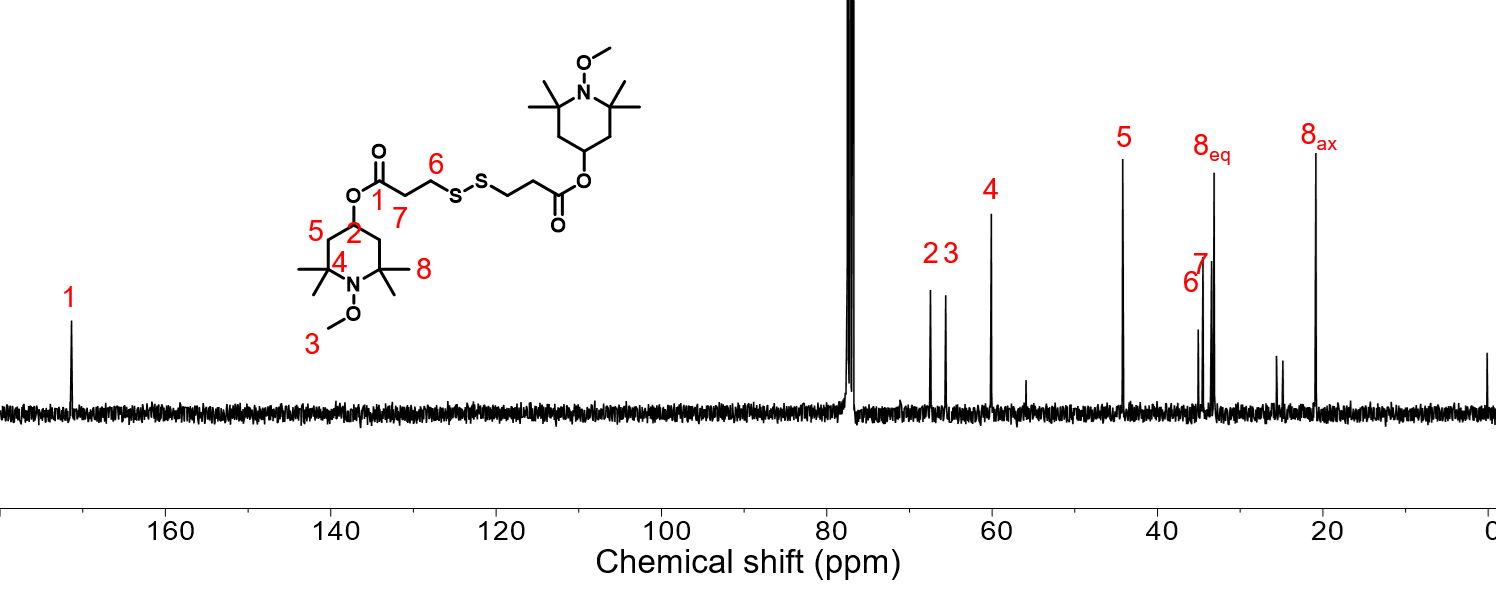


**Figure S4.** ^13^C NMR spectrum of DiMTEMPO-disulfide (101 MHz, CDCl_3_)


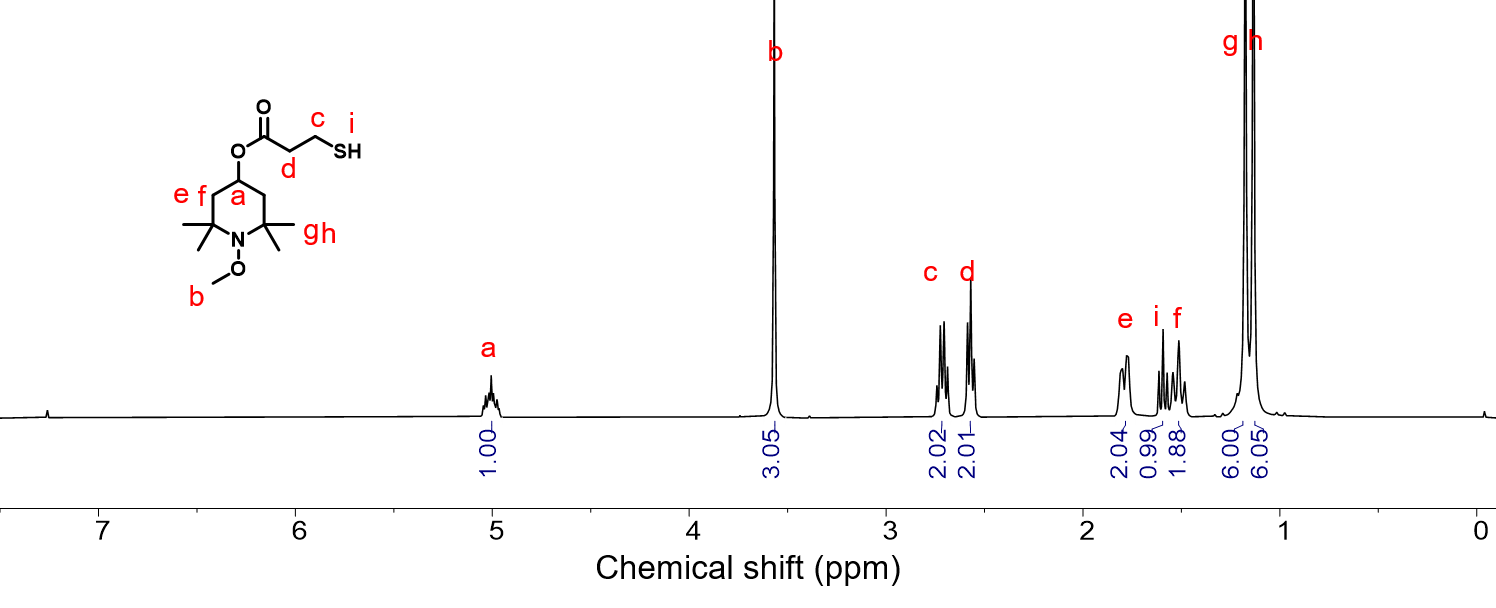


**Figure S5.** ^1^H NMR spectrum of Thio-MTEMPO (400 MHz, CDCl_3_)

*
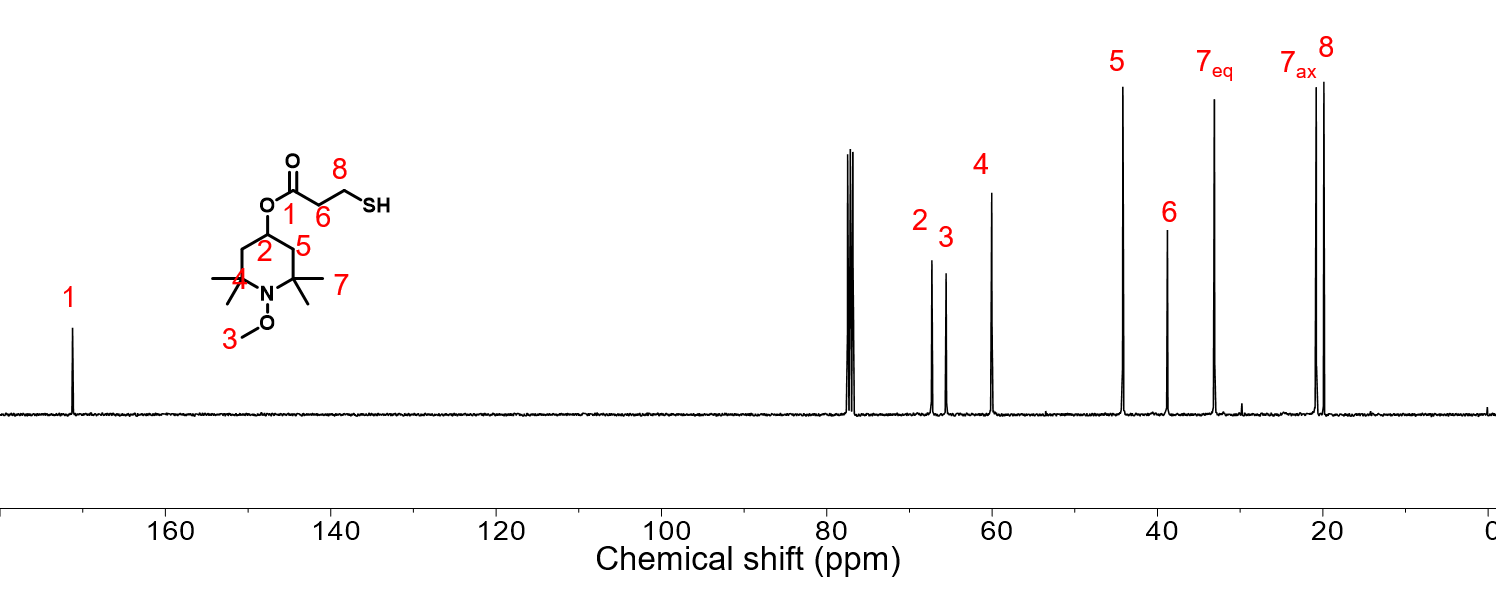
*

**Figure S6.** ^13^C NMR spectrum of Thio-MTEMPO (101 MHz, CDCl_3_)


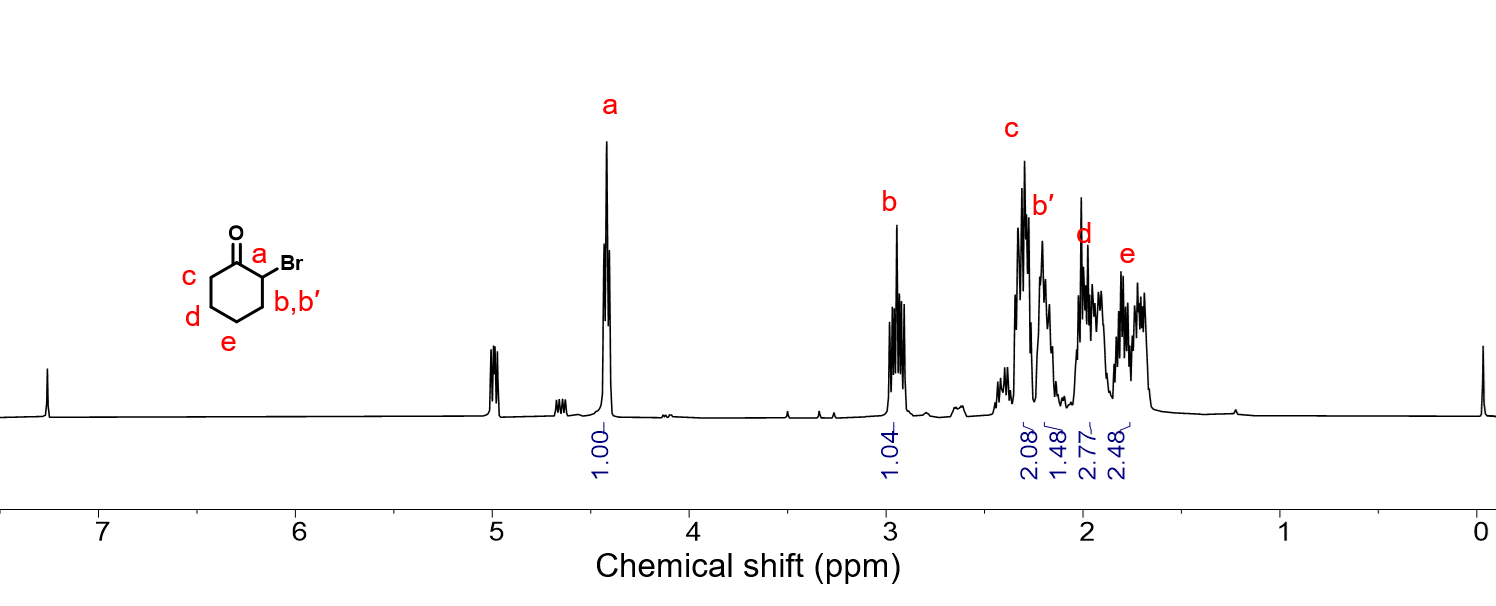


**Figure S7.** ^1^H NMR spectrum of Chex-Br (400 MHz, CDCl_3_)


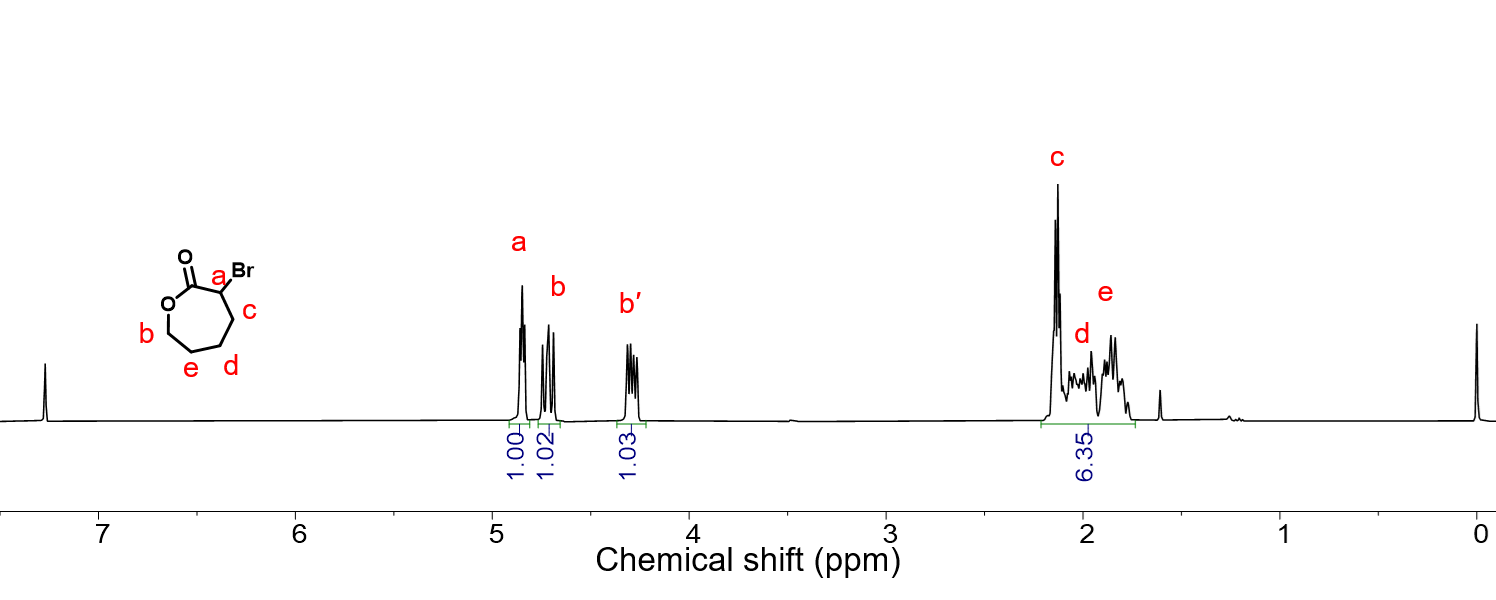


Figure S8. ^1^H NMR spectrum of CL-Br, (400 MHz, CDCl_3_)


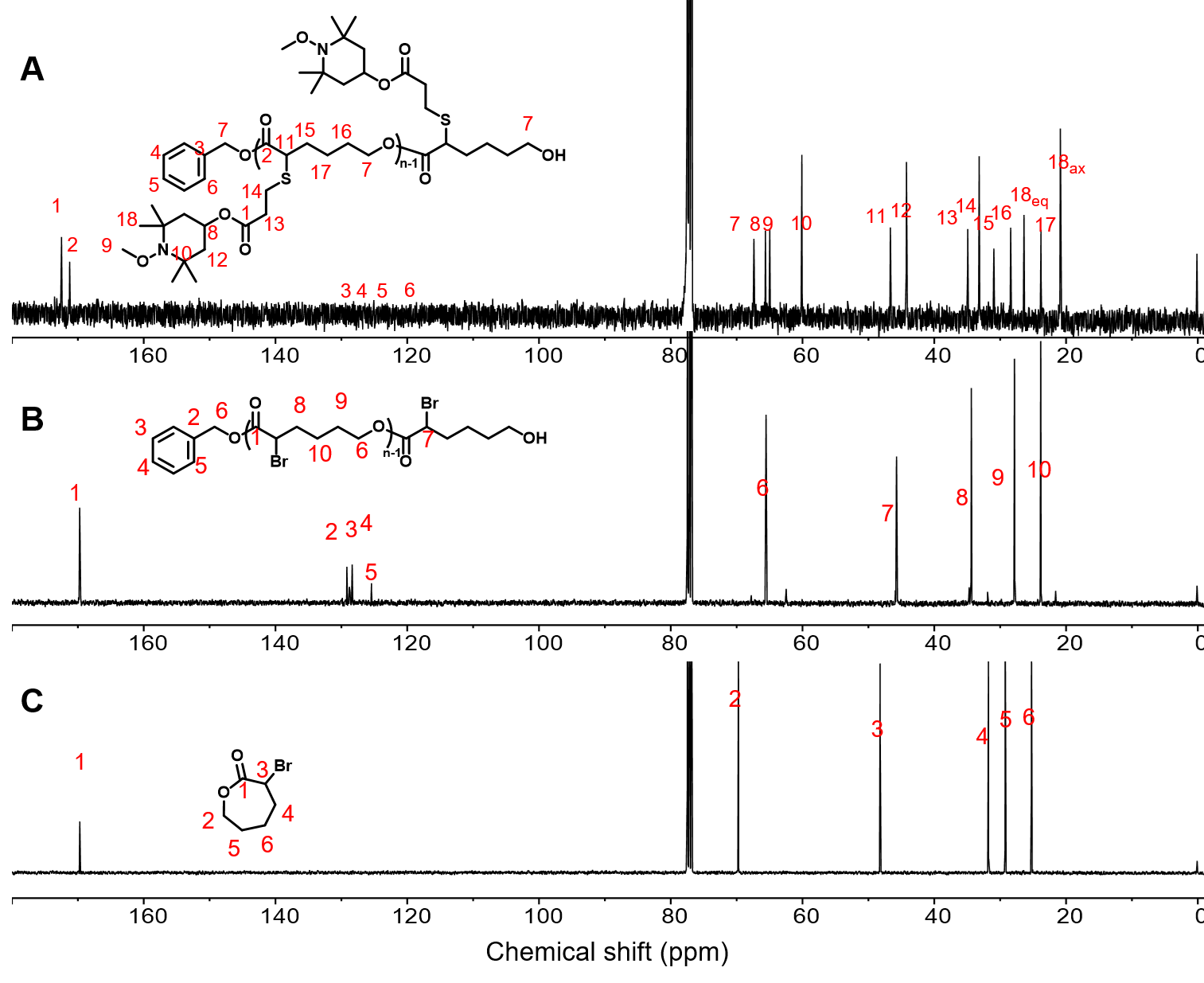


Figure S9. ^13^C NMR spectra of (A) PCL-MTEMPO, (B) PCL-Br, (C) CL-Br, (101 MHz, CDCl_3_)


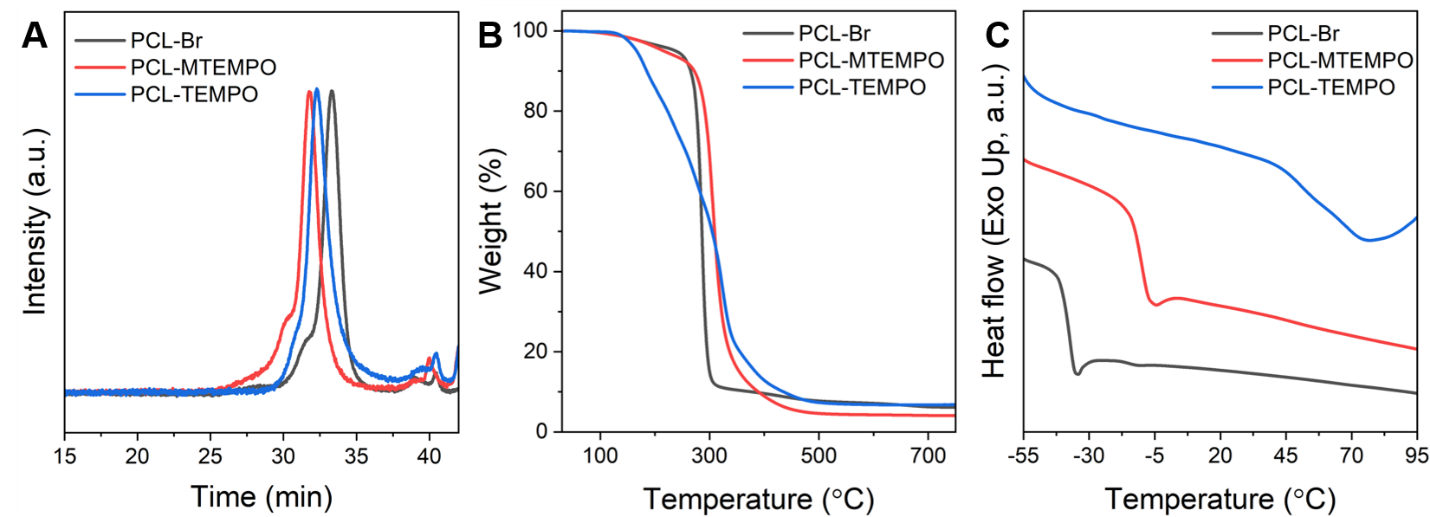


Figure S10. Key physical properties of PCL-TEMPO and its precursors. (A) SEC traces, (B-C) comparisons of thermal properties of PCL-TEMPO and its precursors, for (B) *T*_d_ and (C) *T*_g_.


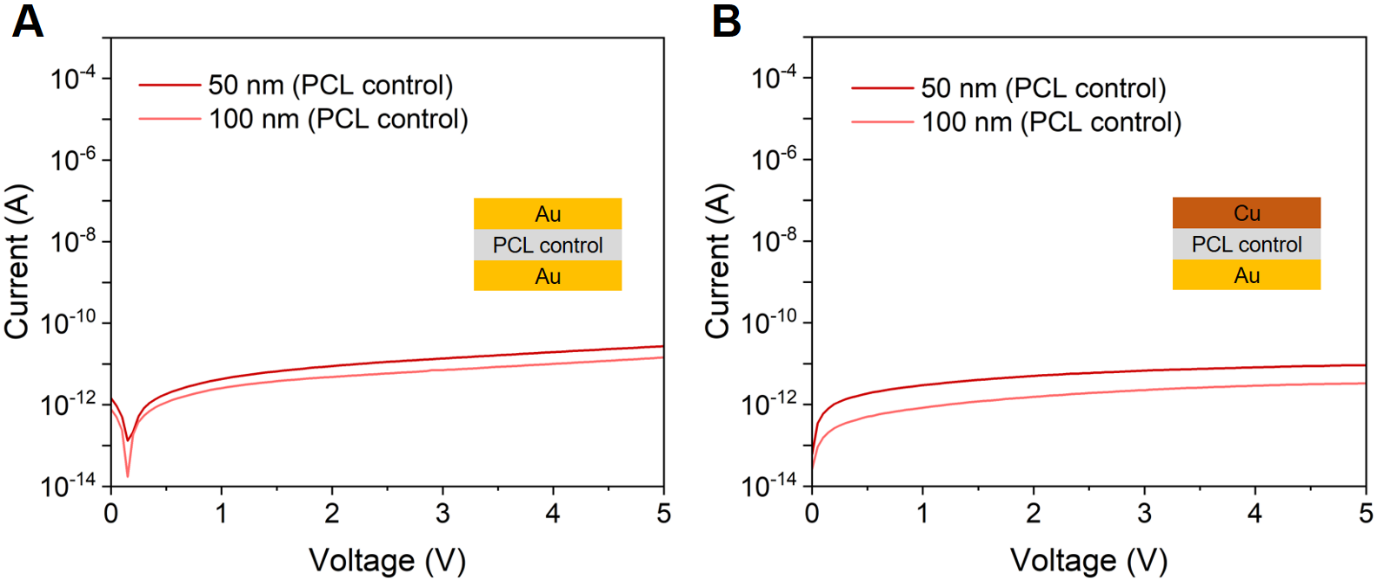


**Figure S11**. Control experiment on resistive switching with PCL as the active layer. (A) *I-V* curves of MIM devices with Au (bottom) / Au (top) electrodes at active layer thicknesses of 50 nm and 100 nm. (B) *I-V* curves of MIM devices with Au (bottom) / Cu (top) electrodes at active layer thicknesses of 50 nm and 100 nm.


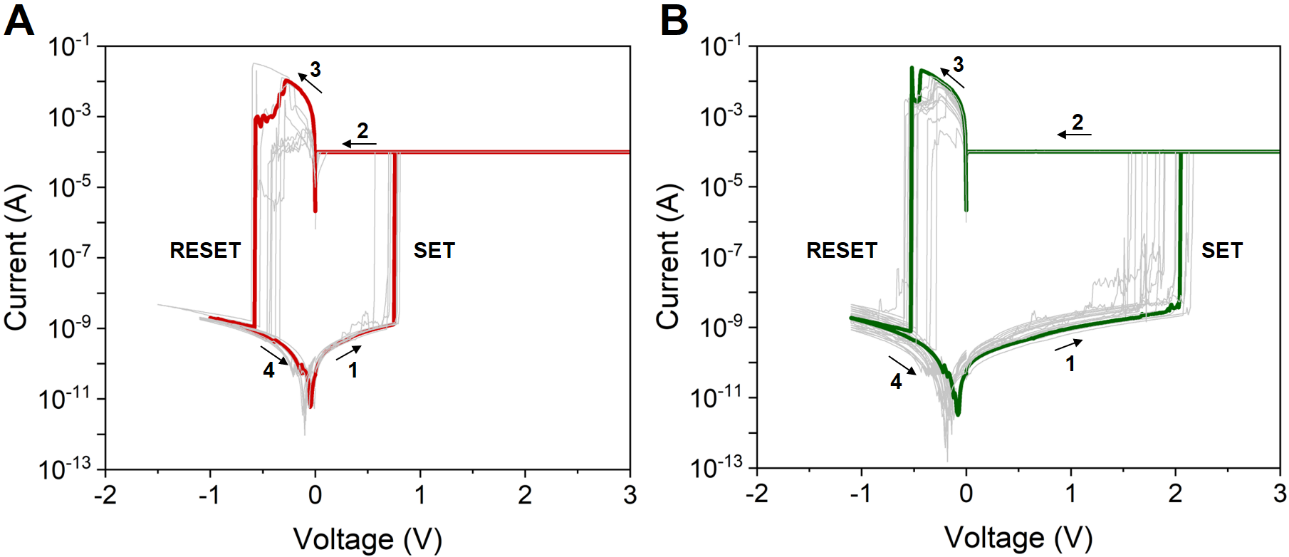


**Figure S12**. Representative *I-V* cycling in a **PCL-TEMPO** based device with (A) a thinner (50 nm) active layer compared to (B) the original (100 nm), showing SET voltage reduction to <1 V.


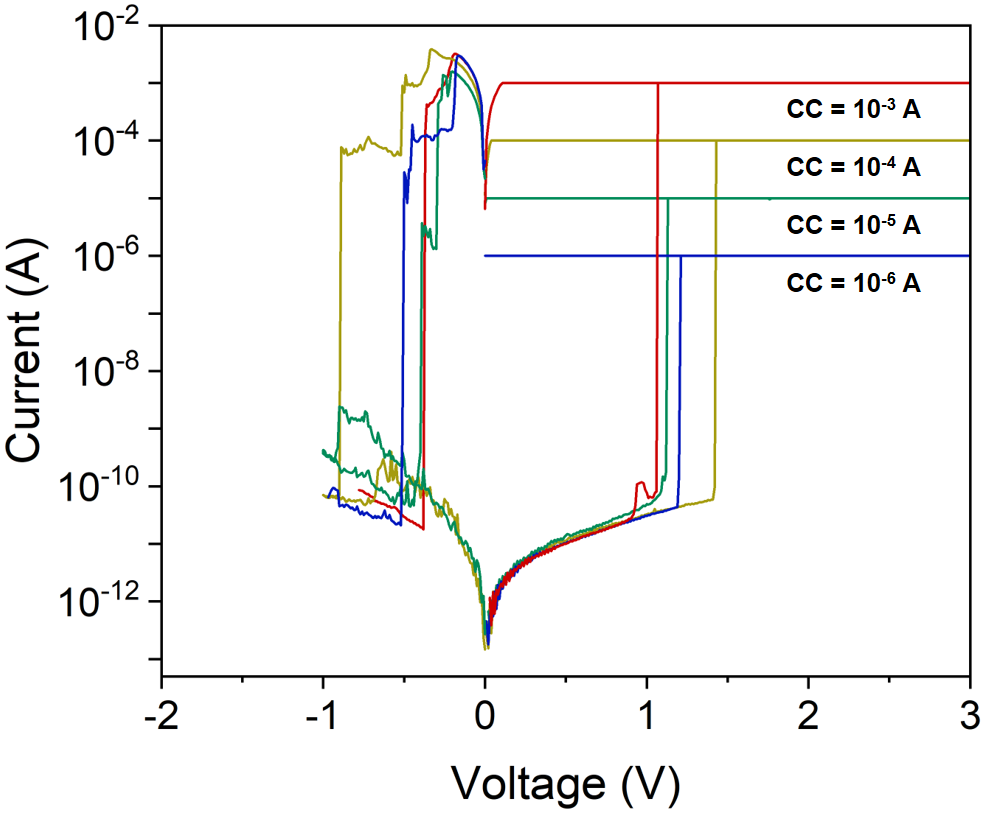


**Figure S13**. Compliance current (CC) independence of resistive switching in **PCL-TEMPO** based device.


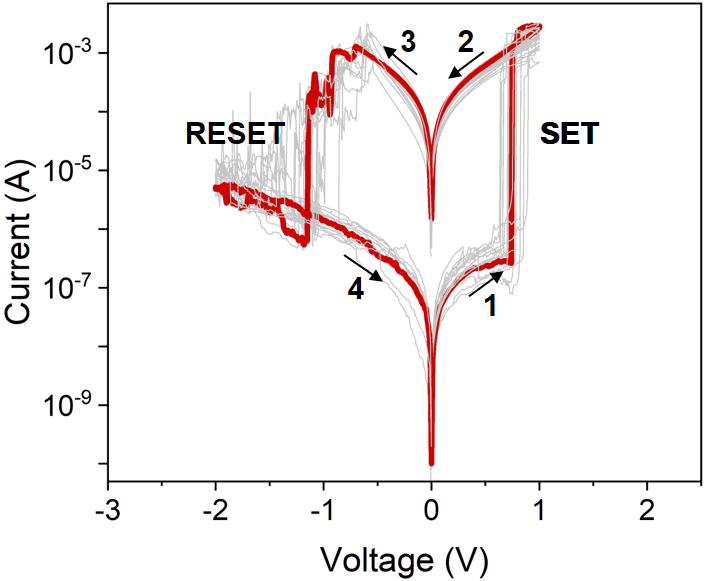


**Figure S14**. Case study on intrinsic resistive switching in a 50 nm-thick **PCL-TEMPO** based device without compliance current.


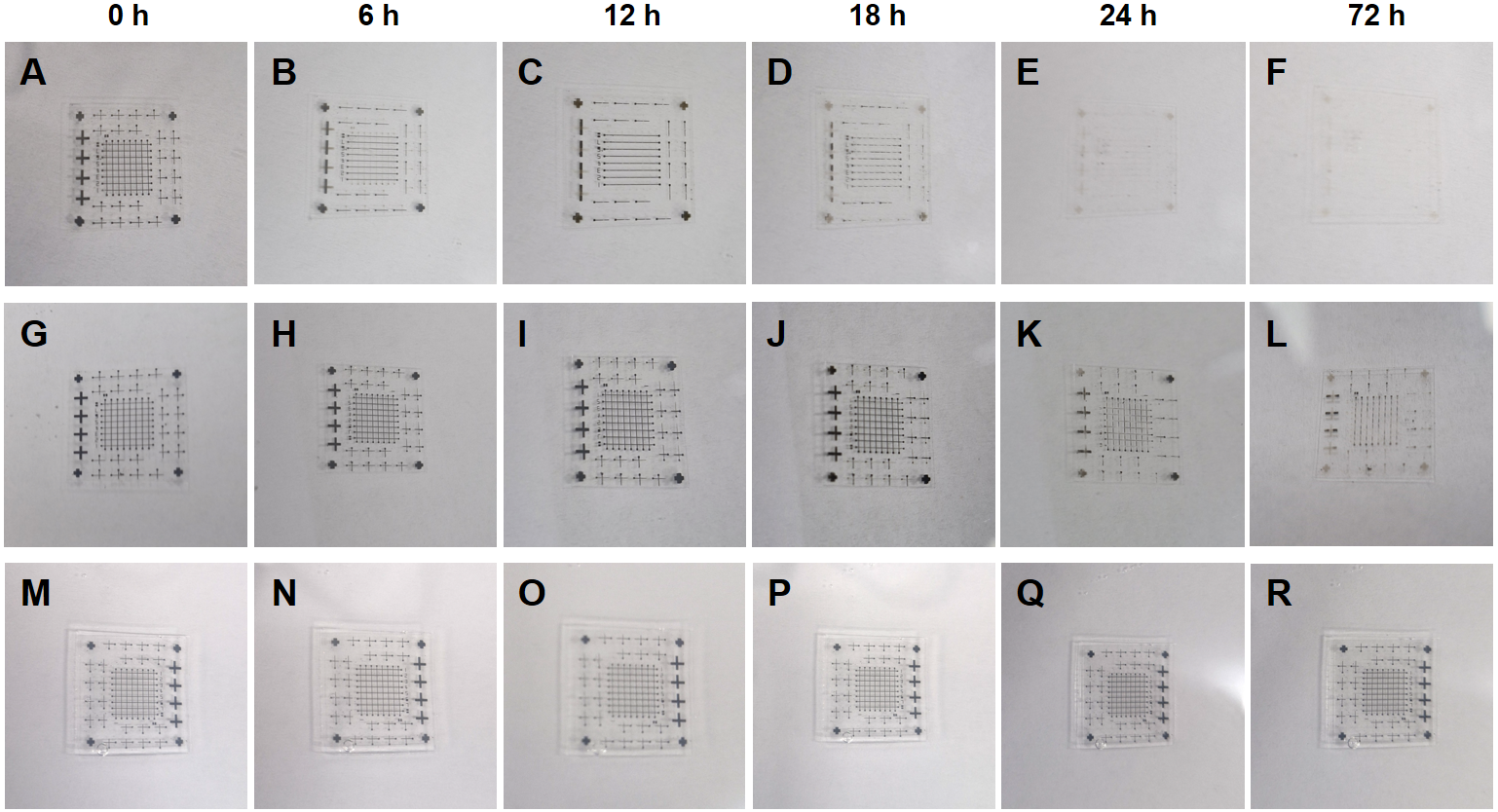


**Figure S15**. Physical transience of the **PCL-TEMPO** devices under different packaging conditions. Photographs showing the devices at different time points in DI water: **(A–F)** a control device as described in the main text, **(G–L)** a device with a PCL passivation layer, and **(M–R)** a device with a PDMS passivation layer. The experimental results clearly demonstrate an additional functionality window enabled by appropriate device packaging technology or engineering. First, the control device (**Figure S15a** to **S15f**) exhibited the first noticeable disappearance of the top electrode within 6 to 12 hours, consistent with the physical transience behavior reported in the original manuscript. However, the device passivated with PCL showed a significant extension of this disappearance time, that begins to occur at 24 hours (**Figure S15g** to **S15l**). Notably, this result suggests that the thickness of the PCL layer can be systematically engineered to further control the degradation kinetics. Lastly, the devices passivated with PDMS exhibited no observable degradation over an extended period, with no noticeable degradation detected even after several weeks of observation (**Figure S15m** to **S15r**).


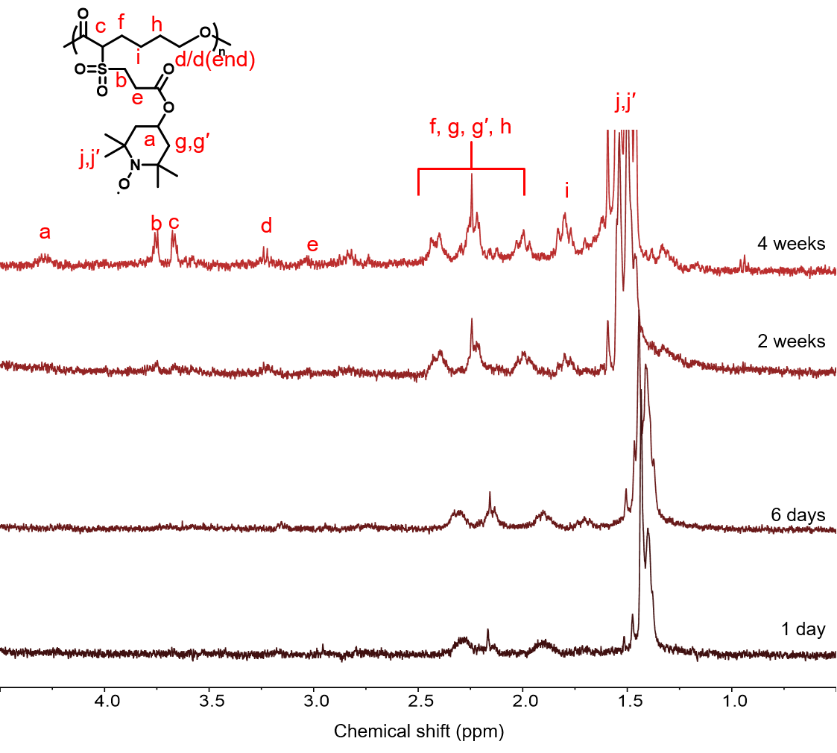


**Figure S16.** ^1^H NMR spectra of **PCL-TEMPO** in D_2_O at 45 ºC after 0.45 μm filtration (400 MHz).


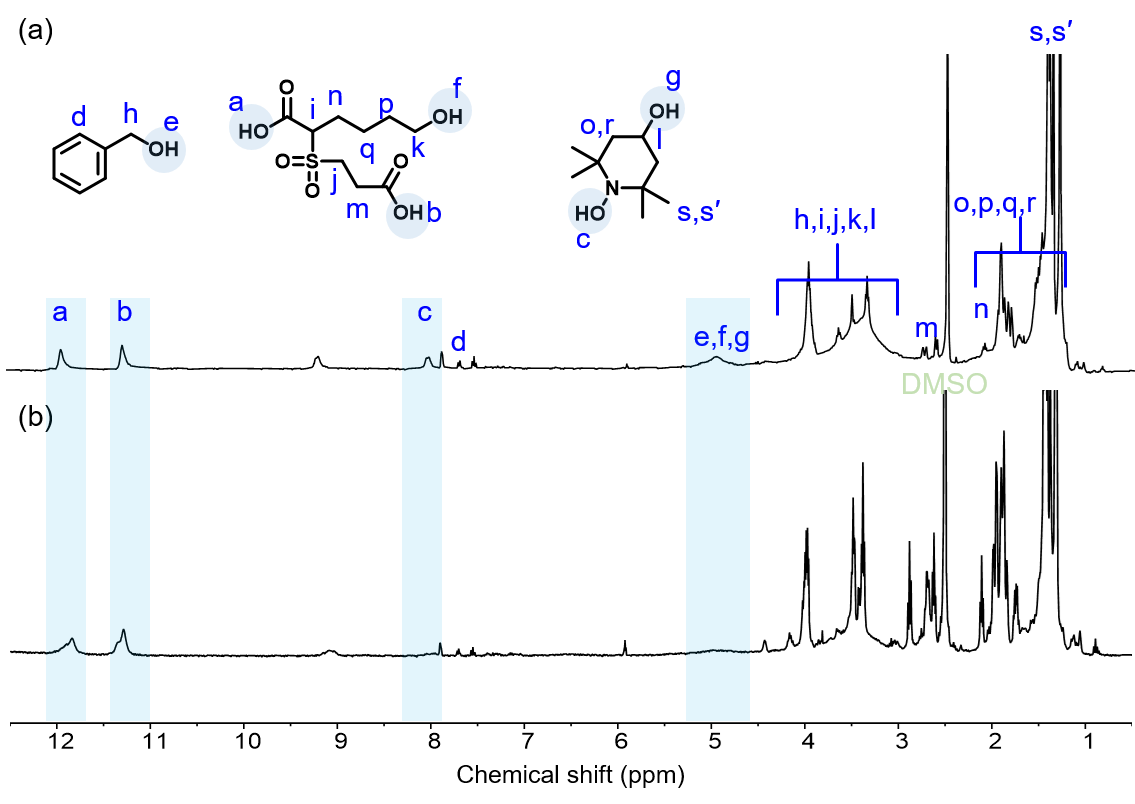


**Figure S17.** ^1^H NMR spectra of **PCL-TEMPO** in DMSO-*d*_6_ after (a) base (ethanol, KOH, 90 ºC, 12 h. Reproduced from ^[64]^ with permission from the Royal Society of Chemistry),^[64]^ and (b) acid (D_2_O, 1M HCl, 80 ºC, 24h) degradation (400 MHz).


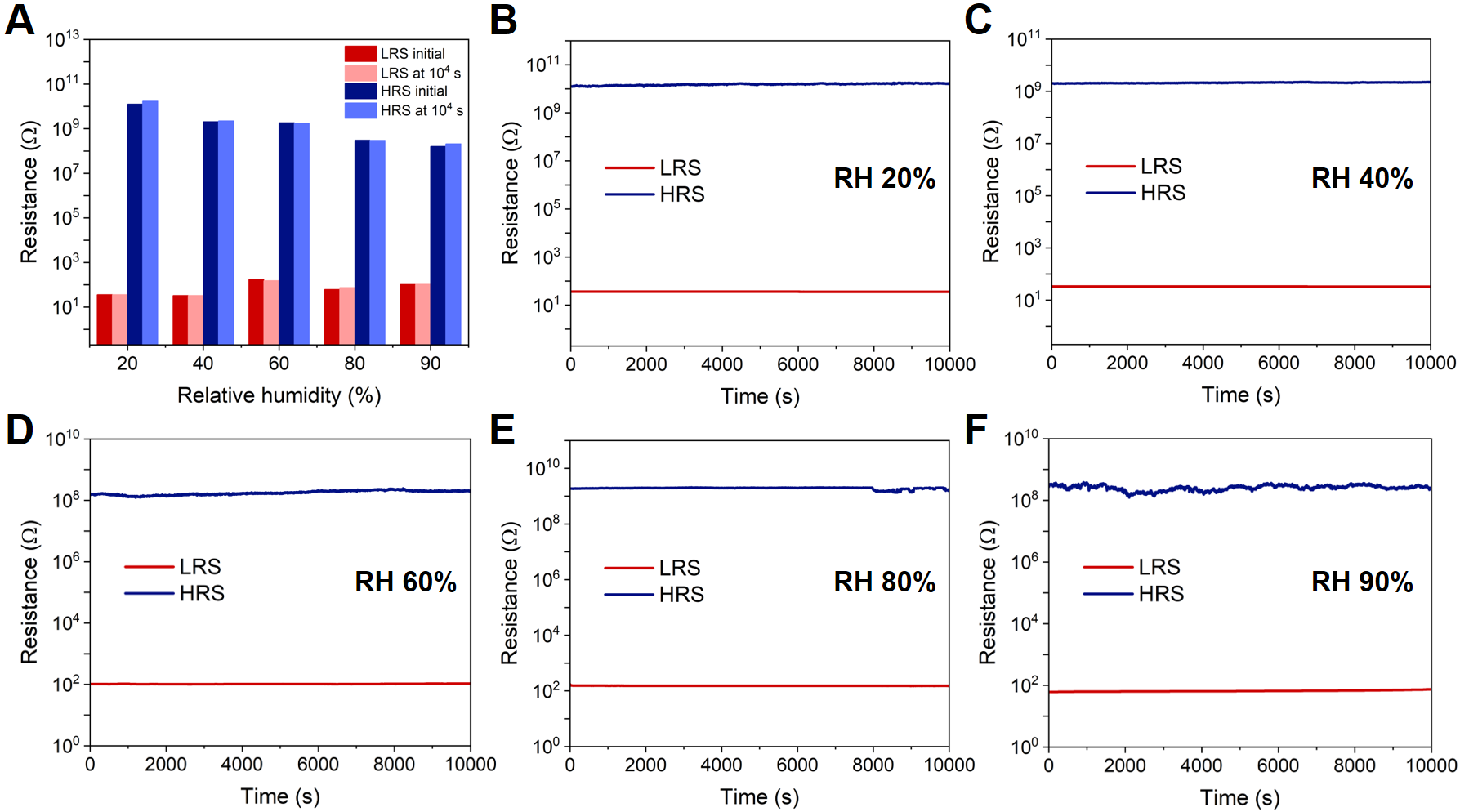


**Figure S18**. Nonvolatile performance degradation test of **PCL-TEMPO** based devices. (A) Summary of the humidity response of LRS and HRS currents at varying RH levels. (B–F) Retention curves showing stable device performance at (B) 20%, (C) 40%, (D) 60%, (E) 80%, and (F) 90% RH.


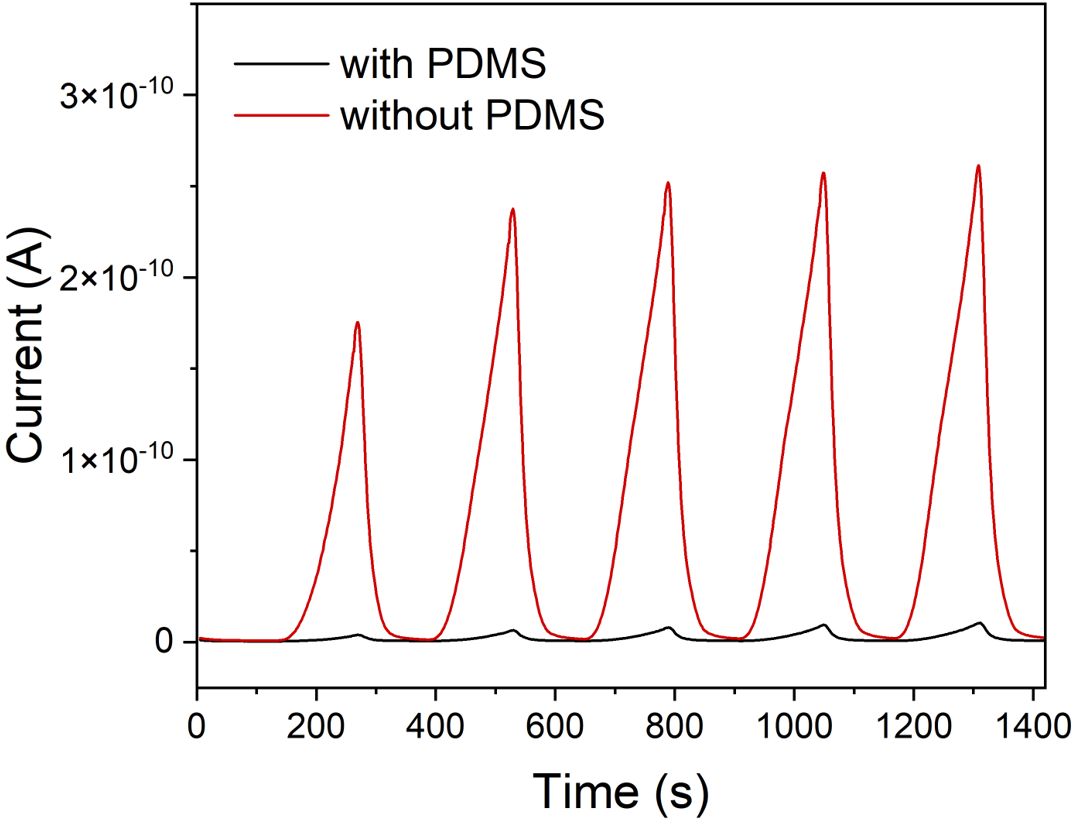


**Figure S19**. Current variation under a series of pulsed water vapor injections at 90% relative humidity, with and without a PDMS passivation layer.

**Table S1**. Molecular weight and thermal properties of PCL-TEMPO and its precursors

|  | *M*_n_ [kg mol^-1^] | *M*_w_ [kg mol^-1^] | *Ð* | *T*_d_ [⁰C] | *T*_g_ [⁰C] |
| --- | --- | --- | --- | --- | --- |
| PCL-Br | 8.8 | 9.9 | 1.12 | 238 | −37.3 |
| PCL-MTEMPO | 14.5 | 18 | 1.24 | 216 | −9.7 |
| PCL-TEMPO | 12.4 | 13.9 | 1.12 | 166 | 50.9 |

**References**

[61] G. Wang, Y. Shi, Z. Fu, W. Yang, Q. Huang, Y. Zhang, *Polymer* **2005**, *46*, 10601.

[62] P. Bexis, A. W. Thomas, C. A. Bell, A. P. Dove, *Polym. Chem.* **2016**, *7*, 7126.

[63] H.-Y. Chen, Y.-L. Lo, P.-L. Wu, P.-C. Lo, L.-F. Wang, *Colloids Surf. B Biointerfaces* **2017**, *156*, 243.

[64] T. Lim, S. Hong, S. Kim, S. Kim, K. Chung, H. Park, Y. Jeong, J.-W. Jeon, J. Chang, S. Cho, *Polym. Chem.* **2015**, *16*, 724
